# Synthesis of Titanium Carbide Powder by a Non-Vacuum Electric Arc Method and Fabrication of Porous Cermets

**DOI:** 10.3390/ma19183925

**Published:** 2026-09-15

**Authors:** Medet Kanapinov, Yernat Kozhakhmetov, Gulnara Yerbolatova, Didar Yeskermessov

**Affiliations:** 1 School of Architecture, Civil Engineering and Energy, D. Serikbayev East Kazakhstan Technical University, Ust-Kamenogorsk 070004, Kazakhstan; 2 Center of Excellence VERITAS, D. Serikbayev East Kazakhstan Technical University, Ust-Kamenogorsk 070004, Kazakhstan; 3 International School of Engineering, D. Serikbayev East Kazakhstan Technical University, Ust-Kamenogorsk 070004, Kazakhstan

**Keywords:** titanium carbide, alloy steel scale, self-propagating high-temperature synthesis (SHS), mechanical activation, porous materials, mechanical properties

## Abstract

Alloy steel scale is a large-tonnage metallurgical waste that represents a potential environmental hazard during storage and disposal. This study investigates its recycling as a component of reactive powder mixtures for producing porous cermets by self-propagating high-temperature synthesis (SHS). Titanium carbide was synthesized from TiO_2_ and carbon using a non-vacuum electric arc method, while the initial powders were mechanically activated to enhance their reactivity and homogeneity. SHS was performed under air, vacuum, and argon atmospheres. The composition of the reactive mixture and synthesis atmosphere significantly affected phase formation, pore structure, and mechanical properties. Within the investigated range, the most favorable combination of structural characteristics was obtained under argon for the composition FeO-45 wt.%, Al-20 wt.%, Al_2_O_3_-7.5 wt.%, Cr_2_O_3_-5 wt.%, TiC-10 wt.%, and FeSi-12.5 wt.%, yielding a total porosity of 60%, an average pore diameter of 121 μm, and a permeability of 48%. SEM and XRD analyses revealed a developed porous structure and Fe–Al, Cr–Al, and Ti–Al–C phases. The cermets exhibited a compressive strength of 18 MPa, flexural and yield strengths of 17 MPa, a hardness of 860 HV, and an elastic modulus of 36 GPa. The developed cermets demonstrate potential for lightweight, permeable, and high-temperature engineering applications while providing an effective route for recycling metallurgical waste.

## 1. Introduction

Metallurgical (industrial) waste represents a significant environmental challenge owing to its large generation volumes and the associated environmental risks. In response to these challenges, increasing attention has been devoted to the development of efficient recycling and resource recovery technologies that facilitate the conversion of metallurgical (industrial) residues into value-added materials, thereby supporting the principles of the circular economy and sustainable resource management [1,2,3,4,5,6,7]. One promising area is waste processing involving the addition of chemical components to produce new functional materials [8,9,10,11,12,13]. Such materials can be used in a wide range of fields, from industrial manufacturing to domestic applications. According to the available literature, there is evidence that porous materials can be produced from industrial waste [14,15,16].

Porous permeable materials have found widespread use in industry, particularly in the purification of air, oils, and fuels, as well as in fuel cells and heating devices that come into contact with high-temperature gas flows. Such materials must possess specific properties, including the ability to operate at high temperatures, heat resistance, and the ability to withstand aggressive environments [17,18].

Open porosity is important when a connection to the interior of the material is required, for example, in filter elements. Open-pored materials can also be used as a matrix for impregnation with molten metal. To overcome these shortcomings, composites with a metal-ceramic matrix were developed. Depending on the desired results, the most commonly used reinforcing materials are ceramic (which improve hardness and heat resistance) or metallic (which improve toughness and flexural strength) [19,20,21,22,23,24,25,26].

Through scientific research, various methods and technological approaches are being developed to create a porous material structure with the required functional and physical-mechanical properties. One of the most promising methods for producing porous metal-ceramics is the replication method using a sacrificial template and direct foaming. The advantage of this method lies in its ability to produce a highly porous material (70–95%) with open pores. The disadvantage of this method is that it requires the use of polymer and ceramic powders to produce the required porous structure [27,28,29,30,31,32,33].

Porous materials with structural and functional properties, produced by spark plasma sintering in an electric field using electric current in various modes depending on whether they are subsequently hot-pressed or cold-pressed, are gaining popularity. The drawback of this method is that it requires a significant amount of electrical energy to produce high-quality material with the desired properties [34,35,36,37].

In addition, the method of low-pressure reactive welding is becoming widely used for the production of porous, intermetallic, and ceramic components from powdered materials. The experiment revealed that rapid heating of a loosely packed mixture results in the formation of an intermetallic compound, accompanied by a reduction in the number of interstitial pores. In addition, it was found that at a temperature of 700–900 °C, the compressive strength ranged from 53 to 72 MPa [38,39,40,41,42].

Using carbothermal synthesis of silicon carbide from silicon oxide produced by sol–gel technology, a porous ceramic with a porosity of 42–55% was obtained. In the work of Simonenko et al. [43], the carbothermal synthesis of silicon carbide from finely divided SiO_2_-C feedstock (produced using sol–gel technology) was investigated, resulting in the formation of silicon carbide ceramics under these conditions. Their work led to the development of a method for producing porous silicon carbide ceramics, which could be used as an inert support for catalysts and filters for the manufacture of SPS (spark plasma sintering) technology. The porosity of the material produced by this method was 42–55%.

In addition, there are studies devoted to the development of porous ceramic materials based on iron oxide and aluminum oxide matrices reinforced with titanium carbide. These studies mainly focus on the mechanical properties of the composites, namely fracture toughness, flexural strength, and hardness [44]. In the work of other authors [45], it was incorporated into a ZnO matrix, and the study focused primarily on investigating the material’s mechanical and electrical properties. The microstructures and mechanical properties of SiC-Ti_2_C composites were analyzed.

The literature review contains numerous studies devoted to the fabrication of porous materials based on Ni–Al and Ti–Al systems, as well as investigations into the effects of various alloying elements on their structure and properties [46,47]. However, these materials often require relatively expensive alloying additions, which increase the overall production cost. A more economical alternative is the fabrication of porous materials based on Fe-containing oxide–Al systems, including Fe_2_O_3_–Al–Al_2_O_3_ compositions [48,49,50,51,52,53,54,55,56]. In particular, Fe–Al-based cermets produced by self-propagating high-temperature synthesis are of considerable interest because the aluminothermic reduction of iron oxides can simultaneously provide the thermal energy required for combustion synthesis and promote the formation of Fe–Al intermetallic phases within the metallic framework. The use of metallurgical wastes, particularly steel mill scale rich in iron oxides, as a raw material for such systems provides an additional advantage by reducing the consumption of primary raw materials and enabling the conversion of industrial waste into value-added porous products. Among the available processing routes, self-propagating high-temperature synthesis (SHS) is considered one of the simplest, most energy-efficient, and cost-effective methods for producing porous ceramic and cermet materials [57,58,59,60,61]. Previous studies by Kanapinov et al. [57,58] and Tubalov et al. [59,60,61] demonstrated the feasibility of producing porous permeable cermet materials by SHS using metallurgical waste and oxide-containing powder mixtures. Building upon these studies, the present work employs an energy-efficient SHS approach utilizing alloy steel mill scale together with metallic and oxide additives as starting components.

Titanium carbide was selected as the ceramic reinforcing component because of its high hardness, thermal stability, high melting point, and resistance to wear and chemical degradation at elevated temperatures. Compared with many oxide ceramic reinforcements, TiC can provide effective local strengthening of the cermet framework without introducing an additional oxide phase into the metallic matrix. Furthermore, its compatibility with Fe- and Al-containing systems makes TiC a suitable reinforcement for SHS-derived cermets intended for high-temperature applications. In the present system, TiC is expected to contribute to the strengthening and thermal stability of the solid framework, while the metallic and intermetallic phases formed during SHS provide structural continuity and toughness. Therefore, the combination of TiC reinforcement with an Fe–Al-based matrix derived from metallurgical waste offers a promising route for balancing porosity, mechanical strength, and high-temperature stability.

Despite numerous studies on porous materials produced by SHS, the application of plasma-synthesized titanium carbide in reactive powder mixtures based on alloy steel mill scale for the fabrication of porous Fe–Al-based cermets remains insufficiently investigated. In particular, the combined effects of TiC reinforcement, ferrosilicon, oxide additives, and the synthesis atmosphere on phase formation, pore structure development, and mechanical behavior have not been systematically established for such waste-derived SHS cermets. This study addresses this gap by establishing relationships between the processing conditions, phase evolution, porous structure, and mechanical properties of the resulting cermets. Therefore, the aim of the present study is to fabricate porous Fe–Al-based cermets by self-propagating high-temperature synthesis using alloy steel mill scale and plasma-synthesized titanium carbide and to investigate the effects of powder mixture composition and synthesis atmosphere on their phase composition, microstructure, porosity, and mechanical properties.

## 2. Materials and Methods

### 2.1. Sample, Electric Arc Plasma, Mechanical Activation, and SHS Preparation Processes

Experimental studies on the production of titanium carbide from titanium oxide were carried out at the National Research Tomsk Polytechnic University using a three-phase direct-current electric arc reactor (Tomsk, Russia) under ambient conditions [62,63,64,65]. This reactor comprises two main modules: the power supply and the reactor zone. Graphite rods 18 mm in diameter and 200 mm long were connected to the terminals of the current source as the anode, whilst cylindrical crucibles 30 mm in diameter and 30 mm high were connected as the cathode [62,63]. A graphite plate was mounted on the guidepost, into which a graphite crucible (55 mm high and 50 mm in outer diameter) was placed. The inner walls of this crucible were lined with graphite felt, after which a smaller crucible (30 mm high and 30 mm in diameter) was placed inside the main crucible, into which the starting mixture was loaded. To prevent impurities from entering the synthesis product, the small graphite crucible was covered with a graphite lid. Graphite rods (18 mm in diameter and 200 mm in length) were placed inside independent steel sleeves above the inner crucible containing the mixture [66,67].

Titanium dioxide (TiO_2_) powder (OSCh grade, supplied by Special Pure Substances, Moscow, Russia) with an average particle size of 56 µm and industrial-grade carbon black (PAP-808, supplied by Special Pure Substances, Moscow, Russia) with an average particle size of 50 µm were used to prepare the initial mixture. TiO_2_ and carbon were mixed at a stoichiometric molar ratio of 1:3, corresponding to a mass ratio of approximately 2.22:1 (68.9 wt.% TiO_2_ and 31.1 wt.% C), according to the reaction TiO_2_ + 3C → TiC + 2CO. The powder mixture was homogenized in a roller mill under an ambient air atmosphere using a cylindrical metal grinding jar. Ceramic grinding balls with a diameter of 50 mm were used, and the ball-to-powder mass ratio (BPR) was 20:1. Homogenization was carried out for 3 h at a rotation speed of 60 rpm until a homogeneous powder mixture was obtained. The use of ceramic grinding media minimized the risk of metallic contamination during homogenization; however, possible minor contamination originating from the ceramic grinding media was not quantitatively evaluated. The homogenized powder mixture was subsequently placed in graphite crucibles, and graphite rods were used as electrodes for the arc-discharge synthesis. Titanium carbide was synthesized by non-vacuum electric arc plasma treatment using a power supply operating at a voltage of 380 V and a current of 300 A. The arc plasma treatment was carried out for 300 s. After the graphite electrodes have been preheated, the rods are moved to the distance of the gap, and the arc discharges are initiated.

The following powders were selected as the starting materials for the fabrication of porous cermets by SHS: alloy steel scale generated during the heat treatment of metals, aluminum oxide (Al_2_O_3_), aluminum powder (grade ASD-1), chromium oxide (Cr_2_O_3_), ferrosilicon (grade FS-65), and titanium carbide synthesized by the non-vacuum electric arc method. Aluminum oxide, aluminum powder, chromium oxide, and ferrosilicon were supplied by Special Pure Substances (Moscow, Russia). Prior to mixing, all powders were dried in a laboratory drying oven (ShS-80-01 SPU, Smolensk, Russia) at 350–360 K for 3–4 h. The average particle sizes of the starting powders are presented in Table 1.

The scale from alloy steel is a mechanical engineering waste, a by-product of the metal’s heat treatment; consequently, it had to be crushed using a ShD-6 jaw crusher (VIBROTECHNIK LLC, St. Petersburg, Russia) to 1 mm and then ground to 42 µm using an IV-4 vibrating grinding machine (JSC GRANT, Naro-Fominsk, Russia).

The charge reagents were mixed in a roller mill using spherical grinding media, the mass of which accounted for 20–25% of the total mass of all components. The ratio of the mixture to the grinding media was 1:20. The mixing time was 1–2 h to obtain a homogeneous charge.

During a study of mechanochemical activation in a planetary ball mill, laboratory PULVERISETTE-7 classic line (FRITSCH GmbH, Idar-Oberstein, Germany), a series of reactions takes place that influence the state of the mixture and the characteristics of the resulting material, thereby affecting the desired properties. The powders are mechanically activated in air at room temperature using 5 mm diameter steel balls placed in a 45 mm diameter beaker. The mass ratio of grinding balls to powder mixture was 10:1; the processing time varied up to 2 h at a planetary disc rotation speed of 200 to 800 revolutions per minute. Table 2 shows the chemical composition and process parameters for the mechanical activation process.

**Figure 1:**
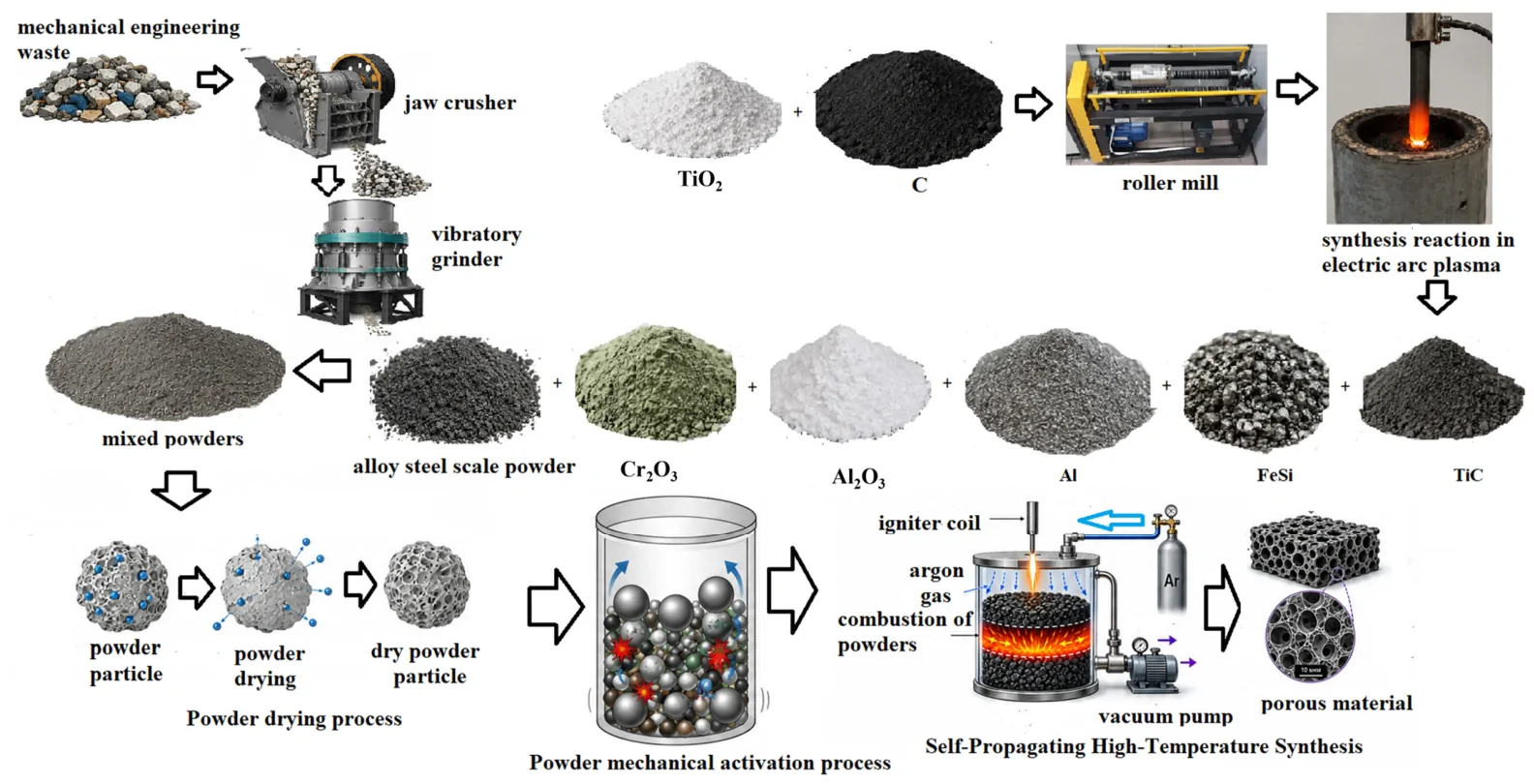
Overall processing route for the fabrication of porous cermet materials from alloy steel mill scale (mechanical engineering waste), including raw-material preparation, mechanical activation, and self-propagating high-temperature synthesis (SHS).

The prepared mixture was poured into the appropriate molds for the production of porous, permeable metal-ceramic materials with a bulk density. The degree of compaction was determined and monitored on the basis of the ratio. Figure 1 shows the process flow diagram for the production of the porous material.

Metal heat treatment waste is crushed in a jaw crusher, after which the material is fed into a vibrating mill to produce a powder. The particle size of the powder was monitored using a particle analyzer. Titanium oxide and carbon powders were mixed in a roller mill until homogeneous, after which they were placed in a graphite crucible for the synthesis of titanium carbide by the electric arc discharge method in the ambient atmosphere. Next, the prepared powders of aluminum, aluminum and chromium oxides, ferrosilicon, titanium carbide, and alloyed steel scale were mixed until homogeneous. The powders were then dried in an oven to remove moisture and placed in the beakers of a planetary mill for mechanical activation. The prepared powders were synthesized using the SHS method under ambient, vacuum, and inert atmosphere conditions. Argon (Ar) was used as the inert gas.

The SHS process was carried out in molds into which the charge was placed at its bulk density without any additional compaction. To initiate the combustion reaction and release thermal energy through a chemical reaction, iron-magnesium thermite was used, ignited by an electric arc generated by a nickel-chromium coil. A transformer rated at 110 V and 10 A was used to heat the nickel-chromium coil. The combustion reaction of the charge took place under conditions of frontal combustion in an open environment, a vacuum, and an inert atmosphere (argon), via the transfer of thermal energy from one layer of the charge to another, under conditions of an exothermic reaction involving the heating of the chemical substance by an external source. Once the combustion process had been completed, natural cooling took place. Table 3 shows the reaction synthesis parameters for the self-propagating high-temperature synthesis method. For the subsequent fabrication of porous cermet materials by SHS, powder mixtures mechanically activated under the optimum conditions identified in this study were used. Based on the obtained results, a planetary ball mill rotational speed of 800 rpm and a milling time of 40 min provided the most favorable mechanical activation conditions within the investigated range.

### 2.2. Characterization Methods

Prior to microstructural characterization, the synthesized samples were prepared using standard metallographic procedures. The specimens were sectioned using a Minitom precision cutting machine (Struers ApS, Ballerup, Denmark), followed by grinding and polishing on a LaboPol-5 grinding and polishing system (Struers ApS, Ballerup, Denmark). The polished surfaces were subsequently etched, rinsed, and cleaned to reveal the microstructure.

The phase composition of the synthesized powders and porous cermet materials was identified by X-ray diffraction (XRD) using an X’Pert PRO diffractometer (PANalytical B.V., Almelo, The Netherlands). Diffraction patterns were recorded over a 2θ range of 20–90° with a step size of 0.02°. The obtained diffraction data were used to identify the crystalline phases formed during the synthesis process. Quantitative phase analysis was performed by Rietveld refinement of the XRD patterns, and the relative contents of the identified crystalline phases were expressed in wt.%. This analysis was used to determine the phase composition of the synthesized titanium carbide powder, including the relative fractions of TiC and residual metallic Ti.

The particle size distribution of the synthesized titanium carbide powder was determined using a ANALYSETTE 22 NeXT laser particle size analyzer (FRITSCH GmbH, Idar-Oberstein, Germany). The measurements were performed to evaluate the particle size characteristics of the powder before its incorporation into the reactive powder mixtures.

The microstructure and elemental composition of the synthesized powders and porous cermets were investigated using a VEGA 4 scanning electron microscope (TESCAN, Brno, Czech Republic) equipped with an Xplore 30 energy-dispersive X-ray spectroscopy (EDX) detector (Oxford Instruments, Abingdon, UK). Scanning electron microscopy (SEM) was employed to examine the morphology and pore structure of the synthesized materials, while EDX analysis was used to determine the elemental composition and distribution of the constituent elements.

### 2.3. Determination of Pore Size and Porosity

The porosity of the porous materials was determined using the hydrostatic weighing method in accordance with GOST 18898-89 [68] on disc-shaped specimens with a diameter of 40 mm and a thickness of 10–20 mm. For each investigated synthesis condition, five independently prepared specimens (*n* = 5) were used for the porosity measurements, and the results are reported as mean ± standard deviation (SD). The average pore size was determined metallographically using an ICX41MET universal research microscope (Sunny Optical Technology Co., Ltd., Ningbo, China) operated in reflected-light mode. Pore-size characterization was based on 250 individual pore measurements for each investigated condition. The principle of the hydrostatic weighing method involves measuring the mass of porous samples in air and in a liquid of known density. When determining porosity, the sample is first weighed in air to an accuracy of ±0.005 g. The sample was then impregnated. The impregnation material was petroleum jelly heated to a temperature of 60 °C, into which the sample was immersed. The impregnation process lasted for two hours. After impregnation, the sample was weighed again in air and in water using the same set of scales (when weighing in water, no air bubbles were allowed to form on its surface). The tests were carried out at a temperature of 25 °C. A VIBRA HT-224RCE laboratory balance (Shinko Denshi Co., Ltd., Tokyo, Japan), with a measuring range of 0–500 mg and an accuracy of 0.5 mg, was used for weighing.

The total porosity was calculated using the following formula:

(1) $$P_{T}=\left(1-\frac{m\cdot \rho_{w}}{\left(m_{1}-m_{2}\right)\cdot \rho_{th}}\right)\cdot 100,$$

where *m*—mass of the dry specimen in air, g;

$m_{1}$—mass of the impregnated specimen in air, g;

$m_{2}$—apparent mass of the impregnated specimen in water, g;

$\rho_{w}$—density of water, g/cm^3^; $\rho_{th}$—theoretical density of the fully dense material.

The theoretical density of the fully dense material was calculated according to the rule of mixtures:

(2) $$\rho_{th}=\;{\left(\sum_{i=1}^{n}\frac{\omega_{i}}{\rho_{i}}\right)}^{-1},$$

where $\omega_{i}$—mass fraction of the i-th component; $\rho_{i}$—theoretical density, and n—total number of components.

The permeability of the porous cermet specimens was determined using a custom-built experimental setup based on steady-state air flow through the porous material. The measurements were performed at room temperature using atmospheric air as the working gas. Prior to testing, the specimens were cleaned, dried to constant mass, and their thickness (L) and cross-sectional area (A) perpendicular to the gas-flow direction were measured.

Each specimen was mounted in a sealed holder to prevent air leakage along its lateral surfaces and to ensure that the gas flow passed through the interconnected open-pore network. Air was supplied using a centrifugal pump. The pressures upstream ($P_{1}$) and downstream ($P_{2}$) of the specimen were measured using pressure gauges, while the volumetric air-flow rate (Q) was recorded using a flowmeter. After steady-state flow conditions were reached, the pressure drop across the specimen was calculated as follows:

(3) $$∆P=P_{1}-P_{2},$$

The gas permeability coefficient (k) was calculated according to Darcy’s law:

(4) $$k=\frac{Q\mu L}{A∆P},$$

where k  is the gas permeability coefficient (m^2^), Q  is the volumetric gas-flow rate (m^3^/s), μ  is the dynamic viscosity of air (Pa·s), L  is the specimen thickness (m), A  is the cross-sectional area perpendicular to the gas flow (m^2^), and ΔP  is the pressure drop across the specimen (Pa).

For comparative analysis of the different compositions and synthesis conditions, the relative gas permeability ($P_{rel}$) was calculated by normalizing the permeability coefficient of each specimen to the maximum experimentally measured value within the investigated series:

(5) $$P_{rel}=\frac{k_{i}}{k_{max}}\times 100\%,$$

where $k_{i}\;$ is the permeability coefficient of the investigated specimen and $k_{max}$ is the maximum experimentally determined permeability coefficient among the investigated specimens.

Measurements were performed at several pressure drops. At each condition, at least three volumetric flow-rate measurements were recorded after stabilization of the gas flow, and the obtained values were averaged.

### 2.4. Mechanical Property Measurements

A MIM.4 universal testing machine (GOST LLC, Moscow, Russia) was used to determine the mechanical properties of the material. This equipment enables the generation of reliable experimental data and its graphical representation. This versatile machine provides accurate measurements and complies with modern testing methods. The tests were carried out at a temperature of 20 °C on cylindrical specimens measuring 20 mm in diameter and 20 mm in height. The measurement error for the final dimensions of the specimen did not exceed 0.01 mm. The number of samples used to determine the average value of the mechanical properties must be at least five. When determining the modulus of elasticity in compression, the specimen was loaded with a step-wise increasing stress up to 0.7–0.8 times the stress corresponding to the expected value of the proportional limit; the difference between steps was 0.1.

The modulus of elasticity (*E*), GPa, was calculated using the formula:

(6) $$E=\frac{∆F\cdot h_{0}}{{∆h}_{avg}\cdot A_{0}},$$

where ∆F—load increment, H;

${∆h}_{avg}$—average absolute deformation (shortening) of the specimen under a load of ∆F mm;

$A_{0}$—initial cross-sectional area of the cylindrical specimen, mm^2^.

The mechanical properties $\sigma_{CS}$ were determined using strain gauges with manual and automated data acquisition (analytical and computational processing methods) in accordance with GOST 4651-2014 (ISO 604:2002) [69]. The sample was continuously loaded until destruction. The maximum load preceding the sample’s destruction was taken as $F_{max}$ load, which corresponds to the compressive strength calculated using the following formula:

(7) $$\sigma_{cs}=\frac{F_{max}}{A_{0}},$$

When determining the yield strength, a load capable of withstanding the permissible mechanical stress of plastic deformation in accordance with the formula:

(8) $$\sigma_{ys}=\frac{F}{A_{0}},$$

The specimens were tested for bending on a Gotech AI7000LA (Gotech Testing Machines Inc., Taichung, Taiwan) testing machine using a three-point bending test in accordance with GOST 3882-74 (ISO 513-75) [70]. In accordance with the Test Methods for determining the transverse bending strength, prismatic specimens measuring 4 mm in height, 4 mm in width, and with a support span of 25 mm are used. In this case, the load was applied to the center of the specimen. The flexural strength $\sigma_{fs}$ was calculated using the formula:

(9) $$\sigma_{fs}=\frac{3\cdot F\cdot l}{2\cdot b\cdot h^{2}},$$

where F—load force in N; *l*—distance between the supports in mm;

*h*—height of the cross-section in mm; *b*—width of the cross-section in mm.

The fracture morphology of the synthesized materials was examined using a JSM-6000 (NeoScope) scanning electron microscope (JEOL Ltd., Tokyo, Japan). Before SEM observation, the samples were coated with a thin conductive gold layer to prevent surface charging.

Vickers microhardness measurements were performed using a DuraScan-20 hardness tester (EMCO-TEST, Prüfmaschinen GmbH, Kuchl, Austria) in accordance with ISO 6507-1:2023 [71]. A test load of 0.5 kgf (HV0.5), corresponding to 4.903 N, was applied. Ten indentations were performed for each sample. Considering the highly porous structure of the SHS-derived cermets, the indentations were positioned on sufficiently large and visually dense regions of the solid matrix, avoiding macroscopic pores, pore edges, cracks, and other structural discontinuities that could affect the accuracy of the indentation measurement. The microhardness was calculated from ten valid measurements and is reported as the mean ± standard deviation (SD).

## 3. Results

### 3.1. Titanium Aarbide Synthesis by Non-Vacuum Electric Arc Plasma Treatment

The use of an electric arc discharge makes it possible to control the phase current and thereby influence the characteristics of the resulting product by adjusting the current set on each direct current power source, as well as the duration of the arc discharge’s exposure to the initial mixture. Experiments have previously been conducted in other studies on the production of titanium carbide, with an arc discharge time and ignition time of 300 s at a fixed current of 300 A. As ignition takes place in the open air, the combustion process produces carbon monoxide (CO) and carbon dioxide (CO_2_). The process for producing titanium carbide from titanium oxide is straightforward but involves a multi-stage chemical reaction leading to the formation of the product and consists of the following steps:

(1) Initiation of an electric arc discharge;
(2) Resistive heating of graphite electrodes in short-circuit mode;
(3) Combustion via electric arc discharge to produce a synthetic product;
(4) Extinction of the arc discharge.

Once the graphite electrodes have been heated by resistance in short-circuit mode, the arc discharge begins. This process involves the actual synthesis of titanium carbide:

TiO_2_ + 3C → TiC + 2CO, (10)

Within the reaction chamber, combustion caused by an electric arc creates a high-temperature zone, with the highest temperatures occurring in the arc-contained region. Furthermore, in a number of chemical reactions, specifically the carbothermal reduction of titanium oxide to titanium carbide, a carbon oxidation reaction takes place [65,66,67]. When the electric arc discharge is initiated following the preheating of the crucibles, the temperature of the upper part of the outer crucible rises to between 575 and 1800 °C. As the duration of the arc discharge increases, the surface temperature of the crucible rises.

**Figure 2:**
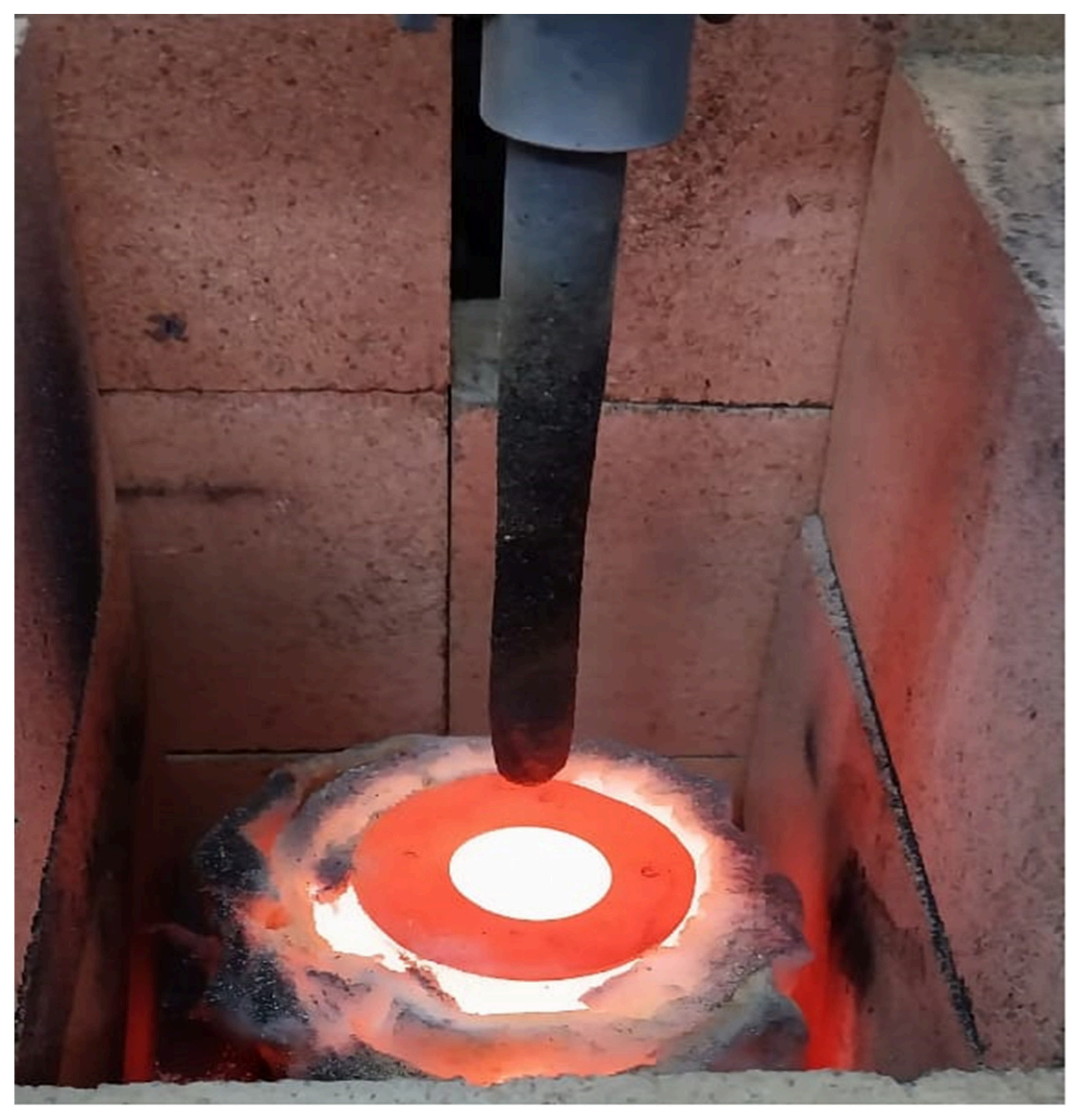
Final stage of titanium carbide (TiC) synthesis by the non-vacuum electric arc method in air, showing the experimental setup and the synthesized product after completion of the arc treatment.

Figure 2 illustrates the final stage of the synthesis process: once the arc discharge ceases, the cathode begins to cool naturally and the thermal field stabilizes, leading to a uniform temperature distribution across the entire crucible surface and the formation of a new phase structure in the form of titanium carbide. The resulting mixture cools and is removed from the apparatus. The total electricity consumption for the entire production process is 0.3 kWh. The results of the analysis of titanium carbide synthesized by the plasma electric arc method under ambient conditions are presented in Table 4. XRD analysis revealed that the synthesized material contained 96 wt.% TiC, confirming a high phase purity of the obtained product. Material losses during the synthesis process amounted to 16%. It should be noted that the reaction was successful and the target product was obtained only upon repeated synthesis, whereas during the first attempt, the process was not fully completed.

**Figure 3:**
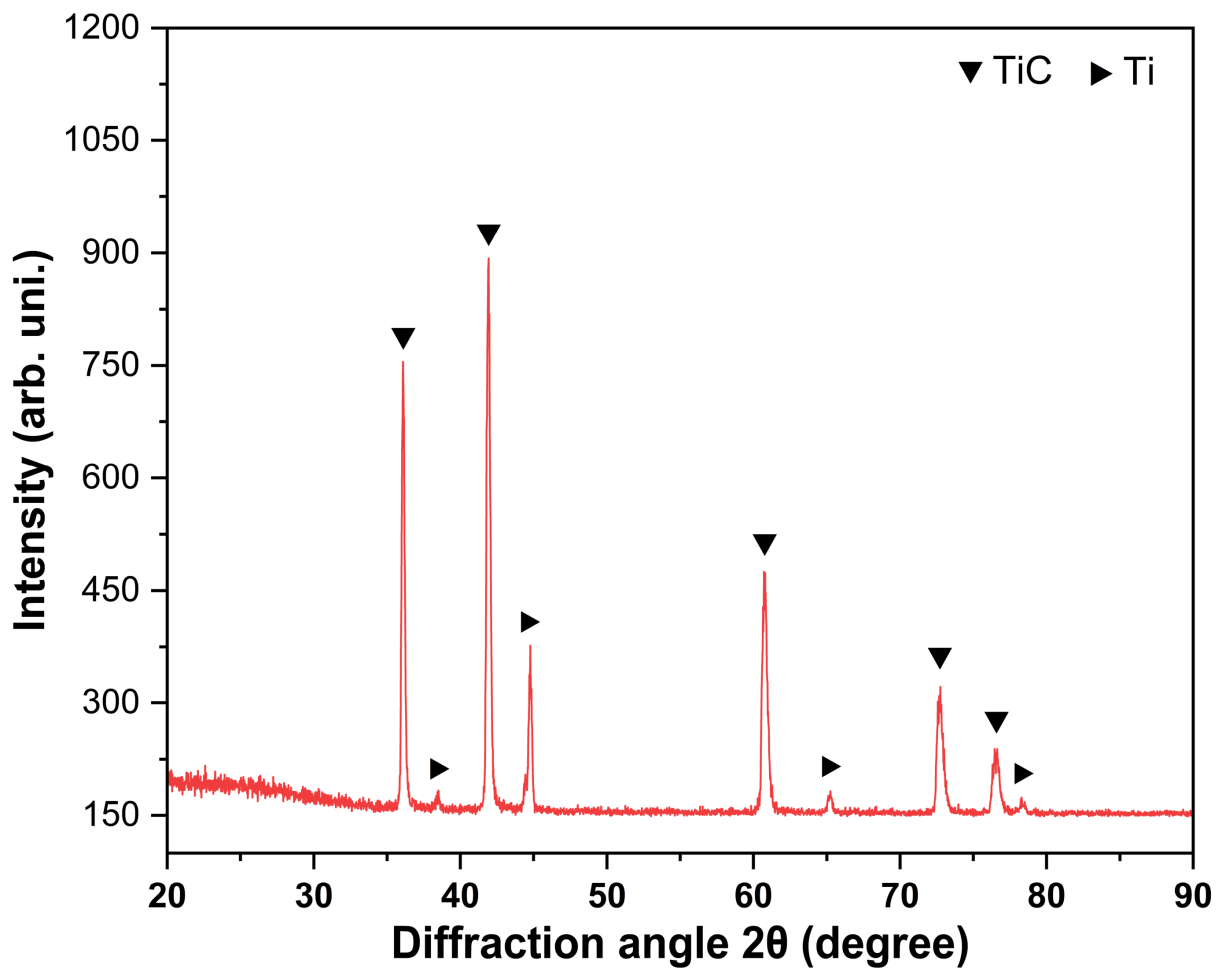
X-ray diffraction (XRD) pattern of titanium carbide (TiC) powder synthesized by the non-vacuum electric arc method in air, showing the phase composition of the synthesized product.

The X-ray diffraction pattern shown in Figure 3 reveals diffraction peaks belonging to the phases of titanium carbide (TiC) and metallic titanium (Ti) in the synthesized sample. The main diffraction peaks correspond to the reference data for the TiC phase with a cubic crystal lattice (space group Fm-3m, No. 225; ICDD Card No. 03-065-4029, PDF-4+ database). In addition, weak diffraction peaks corresponding to the Ti phase, which also possesses a cubic crystal lattice (space group Fm-3m, No. 225; ICDD Card No. 00-069-0392), are observed. Quantitative XRD analysis based on Rietveld refinement showed that the synthesized powder consisted of approximately 96 wt.% TiC and 4 wt.% metallic Ti. The absence of detectable diffraction peaks corresponding to titanium oxide and carbon indicates that these phases were not detected within the detection limits of the XRD analysis, suggesting that the carbide formation reaction proceeded to a substantial extent.

**Figure 4:**
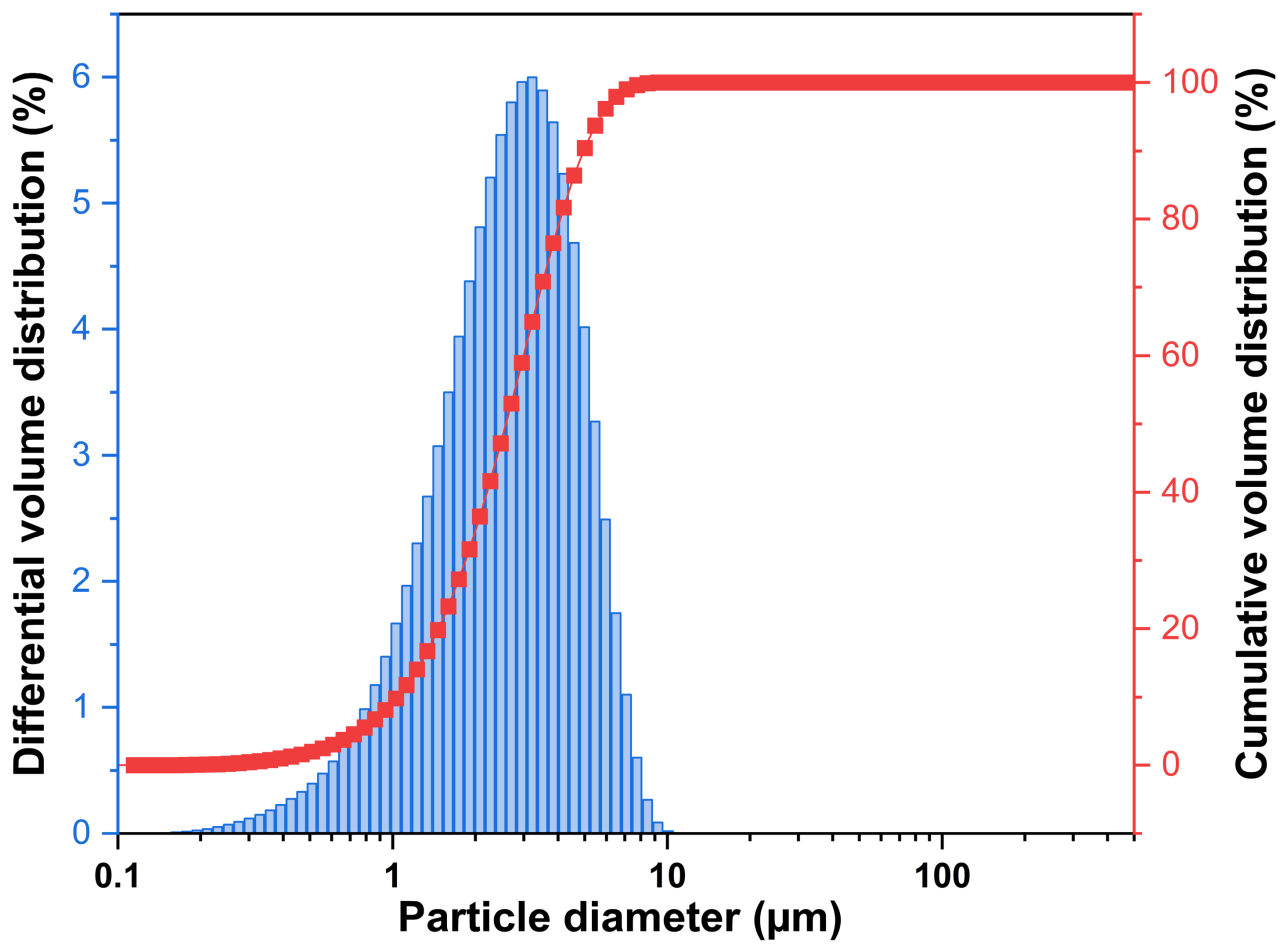
Particle size distribution of titanium carbide (TiC) powder synthesized by the non-vacuum electric arc method in air. The blue bars represent the differential volume distribution (left *y*-axis), while the red line represents the cumulative volume distribution (right *y*-axis).

During studies to determine the particle size and the distribution of the powder using the ANALYSETTE 22 NeXT laser particle analyzer, it was found (see Figure 4) that the mean particle size was 2.558 µm. The size distribution shows the presence of both a fine fraction with particles smaller than 1 µm and larger agglomerates reaching sizes of up to 10 µm. This indicates that the powder investigation has a moderately polydisperse nature, which may affect its flowability, compressibility, and the kinetics of subsequent sintering.

**Figure 5:**
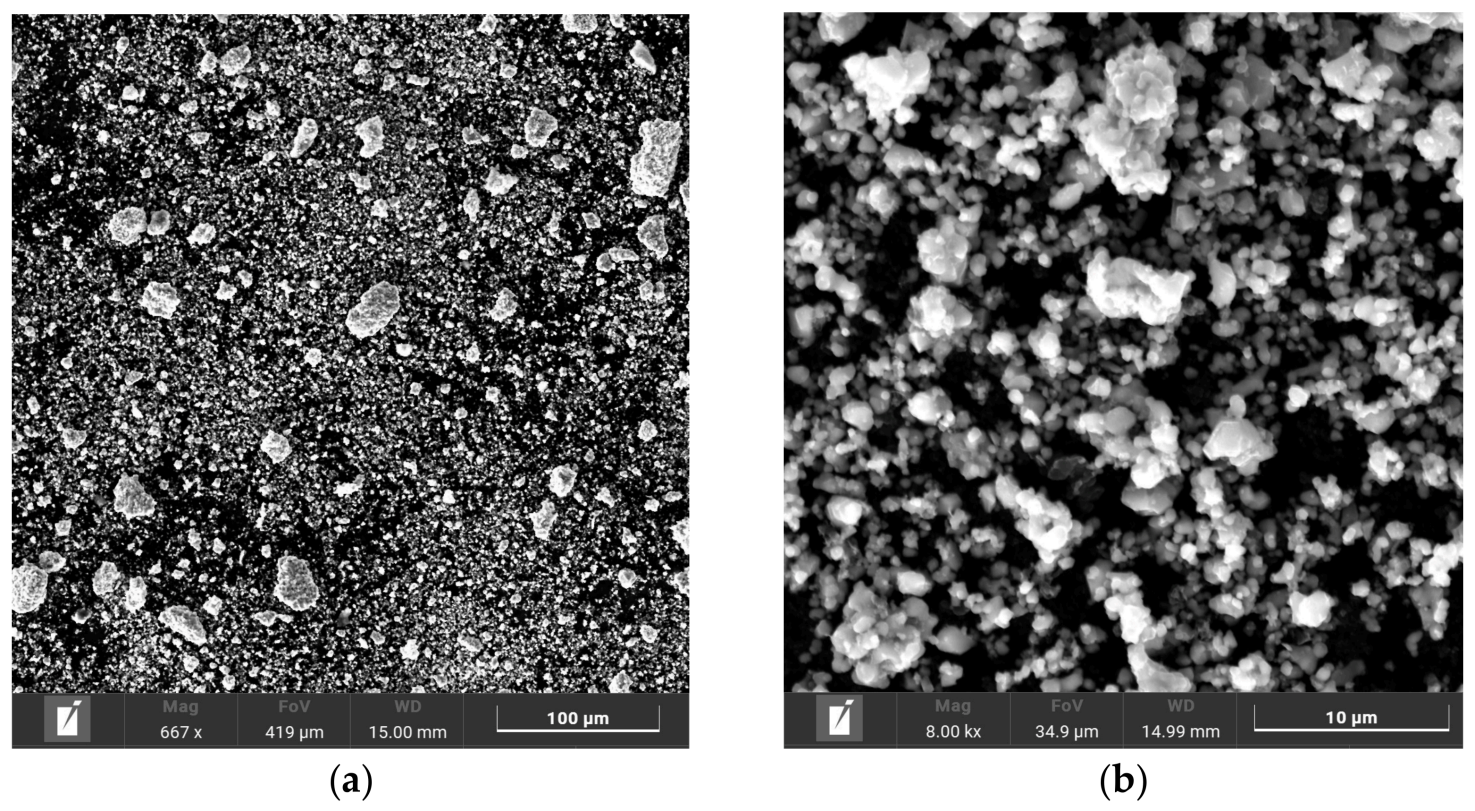
SEM images of titanium carbide (TiC) powder synthesized by the non-vacuum electric arc method in air at different magnifications: (**a**) ×650 and (**b**) ×8000, showing the characteristic morphology of the synthesized TiC particles.

Figure 5 shows the results of scanning electron microscopy (SEM) of a typical sample of the titanium carbide produced.

It was established that the synthesized titanium carbide particles exhibit an irregular morphology with predominantly rounded surfaces and smooth edges. Fine particles exhibit a flaky or plate-like morphology, which is particularly evident at a magnification of ×8000. This morphology is characteristic of carbide powders synthesized at high temperatures and may be attributed to the nucleation and growth mechanisms occurring during electric arc synthesis. The obtained results confirm that the selected arc discharge parameters (I = 300 A, t = 300 s) promote the formation of highly crystalline titanium carbide with a minimal amount of secondary phases and a high conversion degree of the initial reactants.

### 3.2. Effect of Mechanical Activation on the Powder Mixtures

It has previously been established (in the literature) that the sintering kinetics of a multi-component mixture are influenced by the processes of preliminary mechanical activation, depending on the rotation speed and holding time. Research conducted by scientists from various countries has shown that, at high speeds and with shorter retention times, the particle size of the powder changes, making it possible to accelerate the chemical reaction between refractory powders and produce a material with the desired properties. Based on the literature review, it was decided that the powders needed to be activated at high speeds, as the process of saturation occurs due to the grinding of powder particles, which increases the specific surface area and leads to the formation of agglomerates; consequently, composite particles are formed that reduce the specific surface area. However, it was also proposed to conduct the study at low speeds ranging from 200 to 800 revolutions per minute.

**Figure 6:**
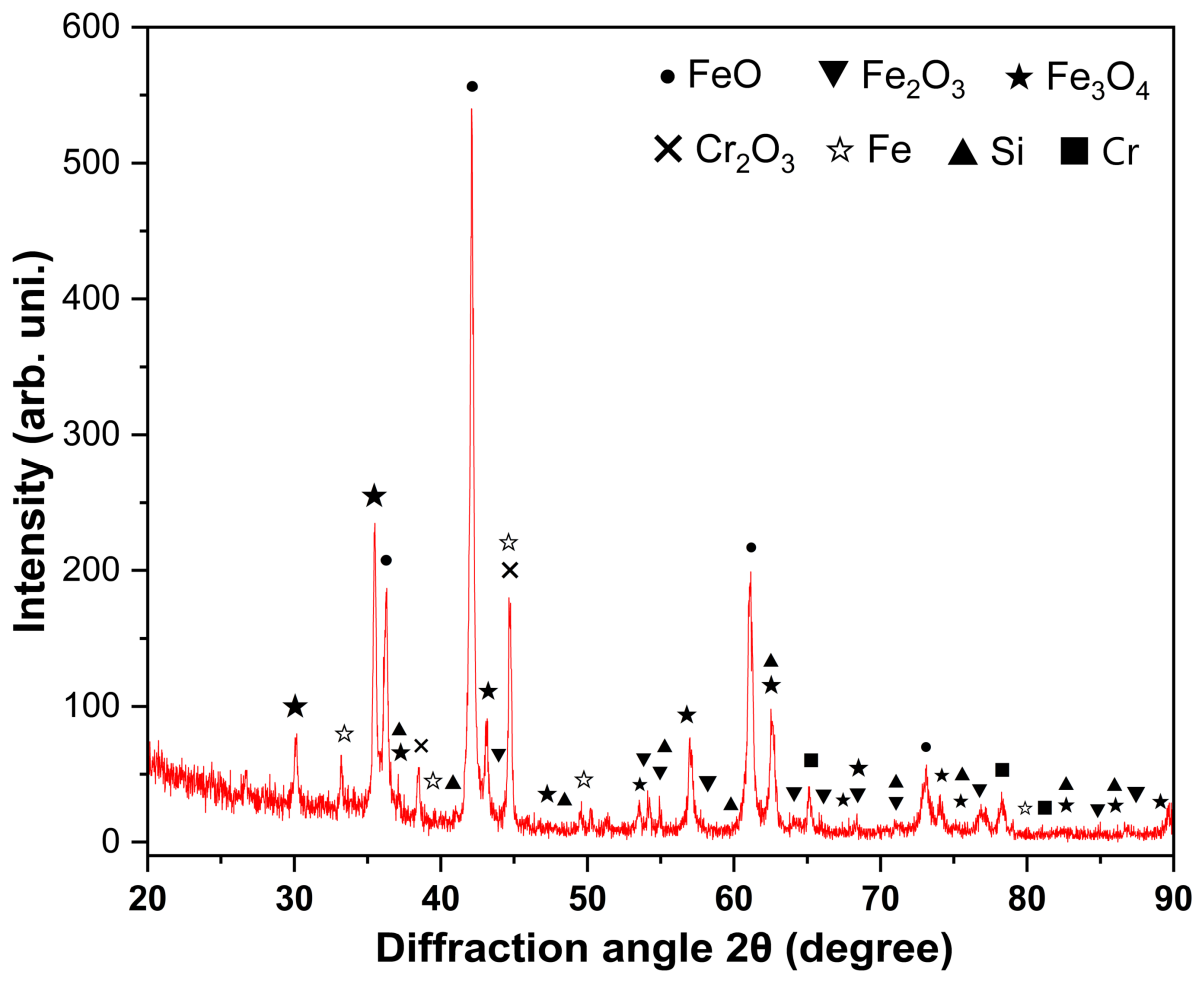
X-ray diffraction (XRD) pattern of the alloy steel scale powder generated during metal heat treatment, showing the phase composition of the initial metallurgical waste used for the preparation of the powder mixtures.

Figure 6 shows the X-ray diffraction pattern of alloy steel scale, which is generated as a by-product during metal heat treatment. The diffraction pattern indicates that FeO and Fe_3_O_4_ are the predominant crystalline phases, whereas Fe_2_O_3_ is represented by less intense reflections, suggesting a lower relative phase fraction.

In addition to the major iron oxide phases, the X-ray diffraction pattern exhibits low-intensity peaks corresponding to chromium and silicon oxides, indicating that alloying elements also participate in oxidation processes under high-temperature conditions. The identified phase composition confirms that the alloy steel scale has a multicomponent structure in which, besides iron oxide phases, the oxides of alloying elements are also present. This should be taken into account when analyzing the mechanisms of oxidation, scale formation, adhesion, and removal in industrial processes.

The reactive powder mixtures consisting of alloy steel scale, aluminum oxide, aluminum, chromium oxide, titanium carbide, and ferrosilicon were mechanically activated in a planetary ball mill at a rotational speed of 800 rpm for 30, 40, and 50 min. Four different powder mixture compositions (designated as Mixtures S 1, S 2, S 3, and S 4) were investigated.

**Figure 7:**
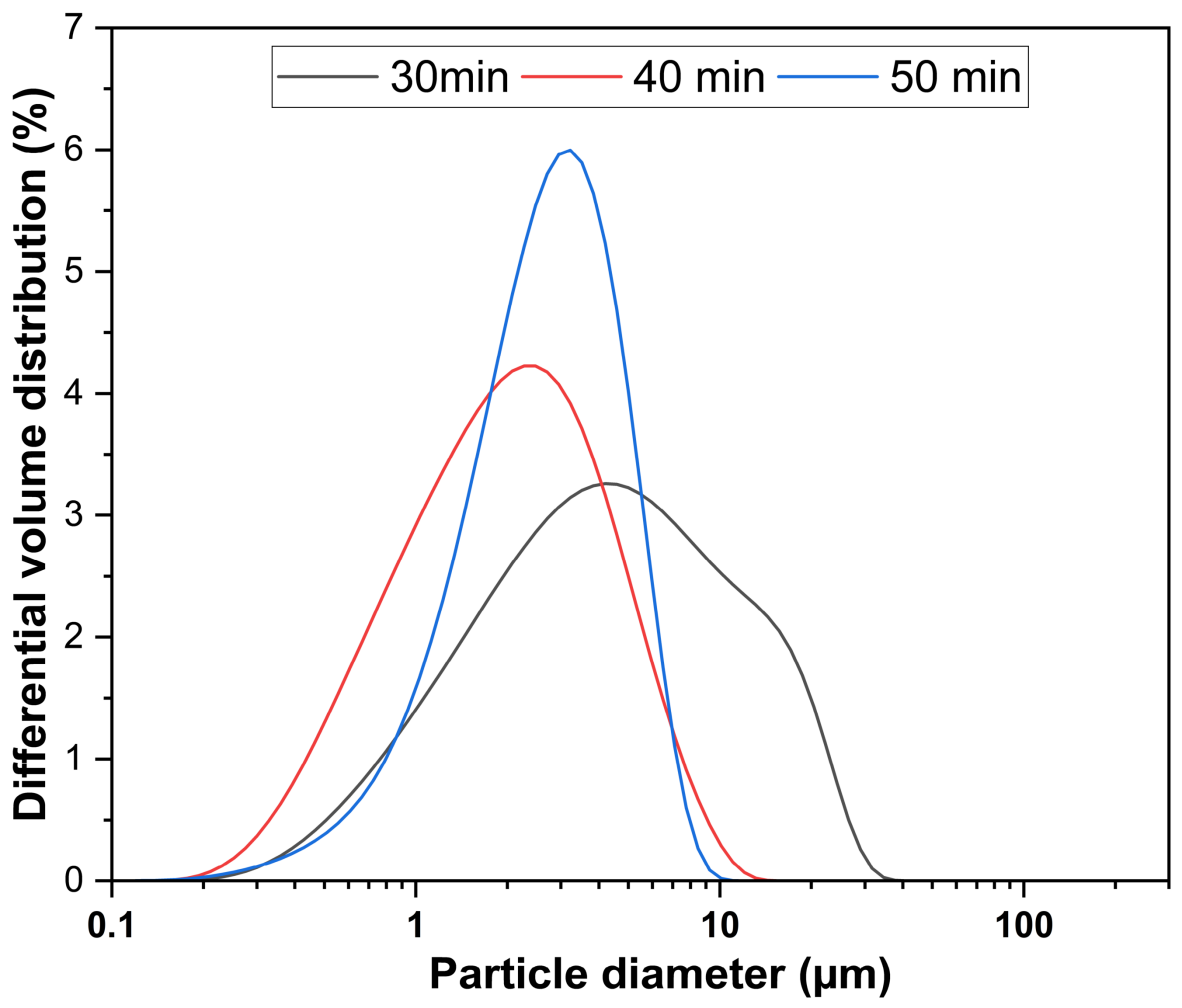
Comparison of the particle size distributions of the S 1 powder mixture after mechanical activation at 800 rpm for different activation times (30, 40, and 50 min), showing the effect of activation time on the particle size distribution.

Figure 7 and Table 5 show the particle size distribution of the powder, expressed as a percentage. The average particle size was calculated on the basis of these results. The average particle size of the powder after 30 min of grinding was 7.34 µm, indicating that a homogeneous structure had been achieved under the specified processing parameters. When the rotational speed was increased to 800 rpm and the process duration to 40 min, the average particle size decreased to 5.8 µm. A further increase in grinding time to 50 min led to a further reduction in the average particle size to 1.3 µm. However, at this stage of the experiment, powder particles were observed to adhere to the walls of the grinding jar, indicating the occurrence of intense mechanization and partial sintering of the particles.

**Figure 8:**
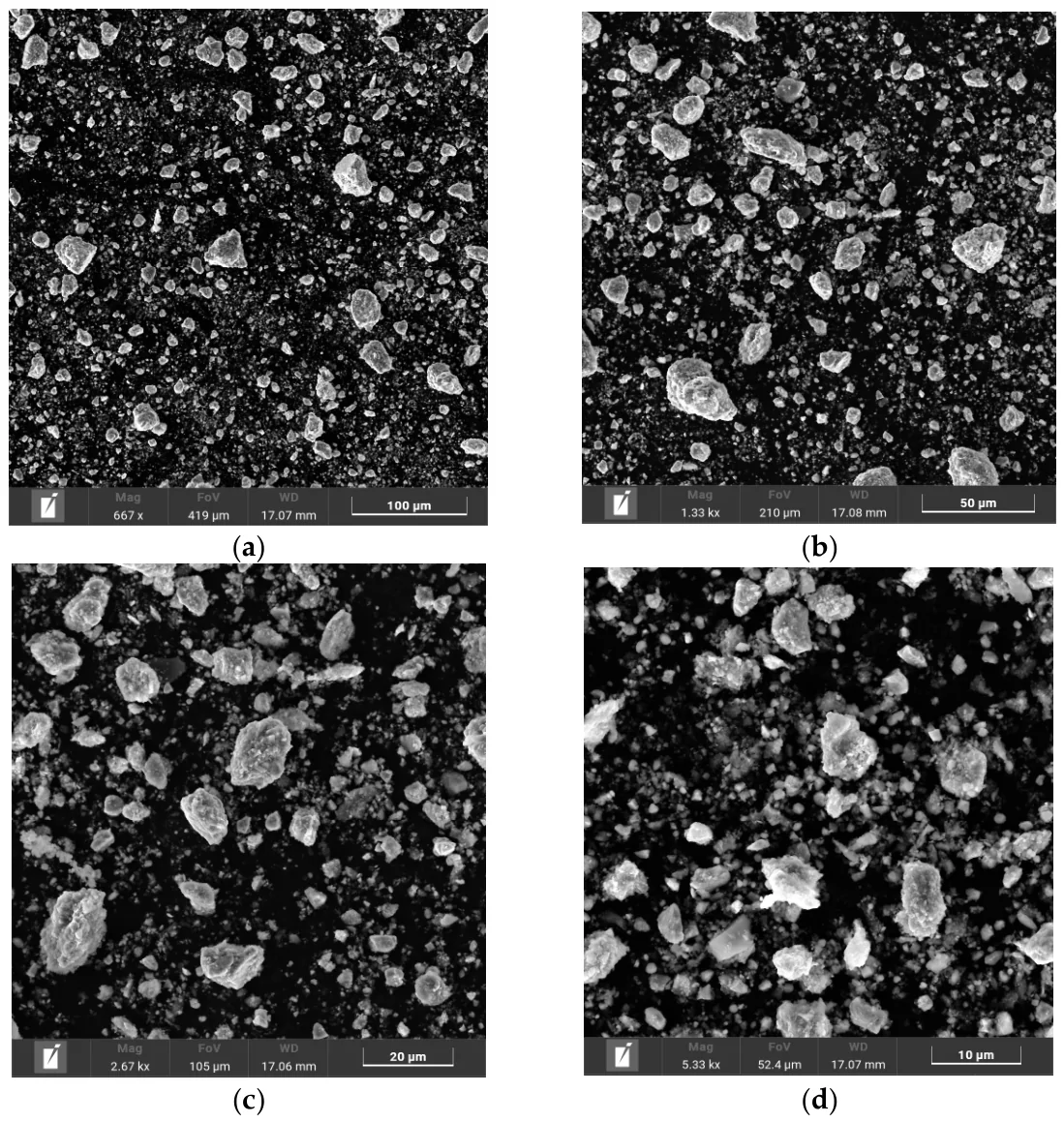
SEM images of the S 1 powder mixture after mechanical activation at 800 rpm for 40 min at different magnifications: (**a**) ×650, (**b**) ×1500, (**c**) ×2500, and (**d**) ×5000, showing the characteristic particle morphology and agglomeration of the mechanically activated powder.

Upon examining the SEM images shown in Figure 8 at ×650 and ×1500 magnification, it becomes clear that oval-shaped particles are distributed throughout the sample. In addition, there is a small number of angular particles. At magnifications of 2500 and 5000 times, scaly particles are clearly visible. These are formed as a result of particles colliding with the grinding balls, which, in turn, is caused by high temperatures and pressures leading to melting and the formation of new phases of iron, silicon, silicon oxide, and aluminum oxide as a result of a reduction reaction. White aluminum particles and gray aluminum oxide particles are also visible. The particles consist mainly of iron and its oxides. In addition, there are agglomerates of large particles, shown in the particle size distribution graph, which are capable of remaining unground during mechanosynthesis in the form of large flakes.

**Figure 9:**
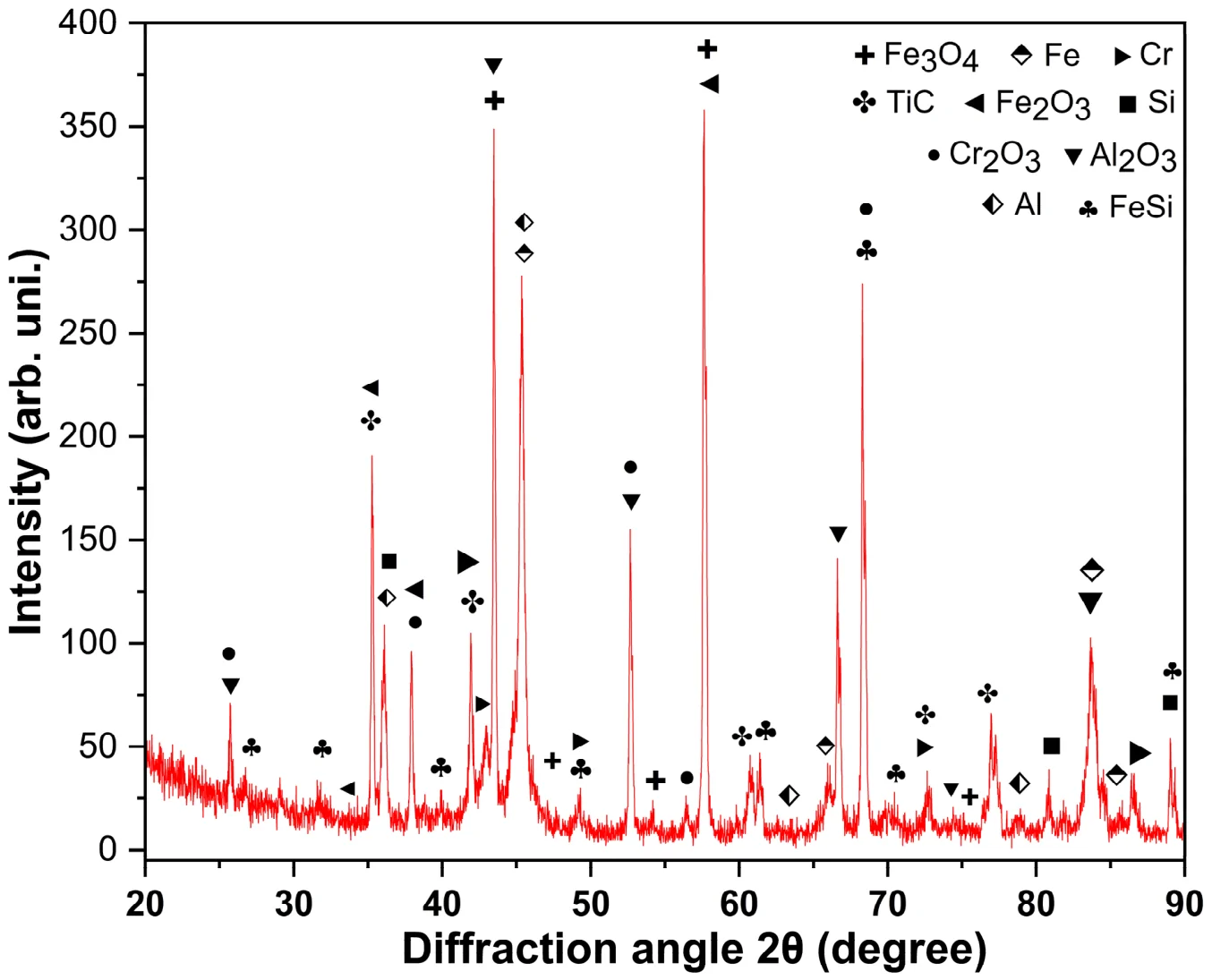
X-ray diffraction (XRD) pattern of the S 1 powder mixture after mechanical activation at 800 rpm for 40 min, showing the phase composition of the mechanically activated mixture prior to SHS.

Figure 9 shows the X-ray diffraction pattern of charge S 1, obtained following the mechanical activation of the powder mixture. The mechanochemical processes occurring during grinding are accompanied by changes in the equilibrium constants of the reactions between the system’s components. As the temperature rises, iron is reduced from iron oxide through interaction with aluminum according to the reaction:

Fe_2_O_3_ + 2Al → 2Fe + Al_2_O_3_, (11)

The occurrence of these reactions is confirmed by changes in the intensity of the corresponding diffraction peaks—an increase in the intensity of the iron and aluminum oxide lines is accompanied by a decrease in the peaks of iron and chromium oxides. This indicates the occurrence of reduction processes and the formation of secondary oxide phases. Thus, during mechanostimulation, both mechanosynthesis processes and reduction reactions take place, caused by the diffusion of atoms of one element into the crystal lattice of another. The results obtained confirm that mechanostimulation is an effective method for the preliminary activation of components for the subsequent synthesis of composite materials.

**Figure 10:**
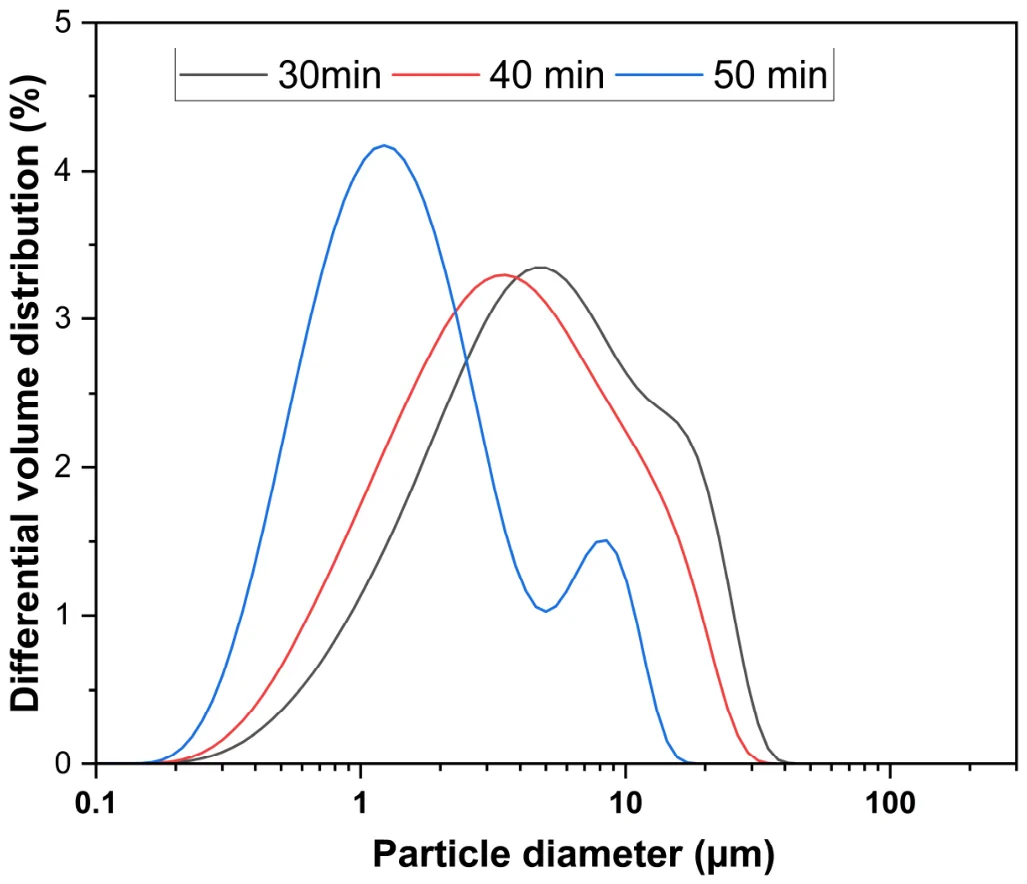
Comparison of the particle size distributions of the S 2 powder mixture after mechanical activation at 800 rpm for different activation times (30, 40, and 50 min), showing the effect of activation time on the particle size distribution.

Figure 10 and Table 6 show the particle size distribution of the powder, expressed as a percentage. The grinding process resulted in the formation of a heterogeneous dispersion system with a uniform distribution of components throughout the mixture. The average particle size of the powder after 30 min of grinding was 6.8 µm, indicating that a uniform structure had been achieved under the specified processing conditions. When the rotation speed was increased to 800 rpm and the process duration to 40 min, the average particle size decreased to 4.4 µm. A further increase in the grinding time to 50 min resulted in a further reduction in the average particle size to 2.45 µm.

**Figure 11:**
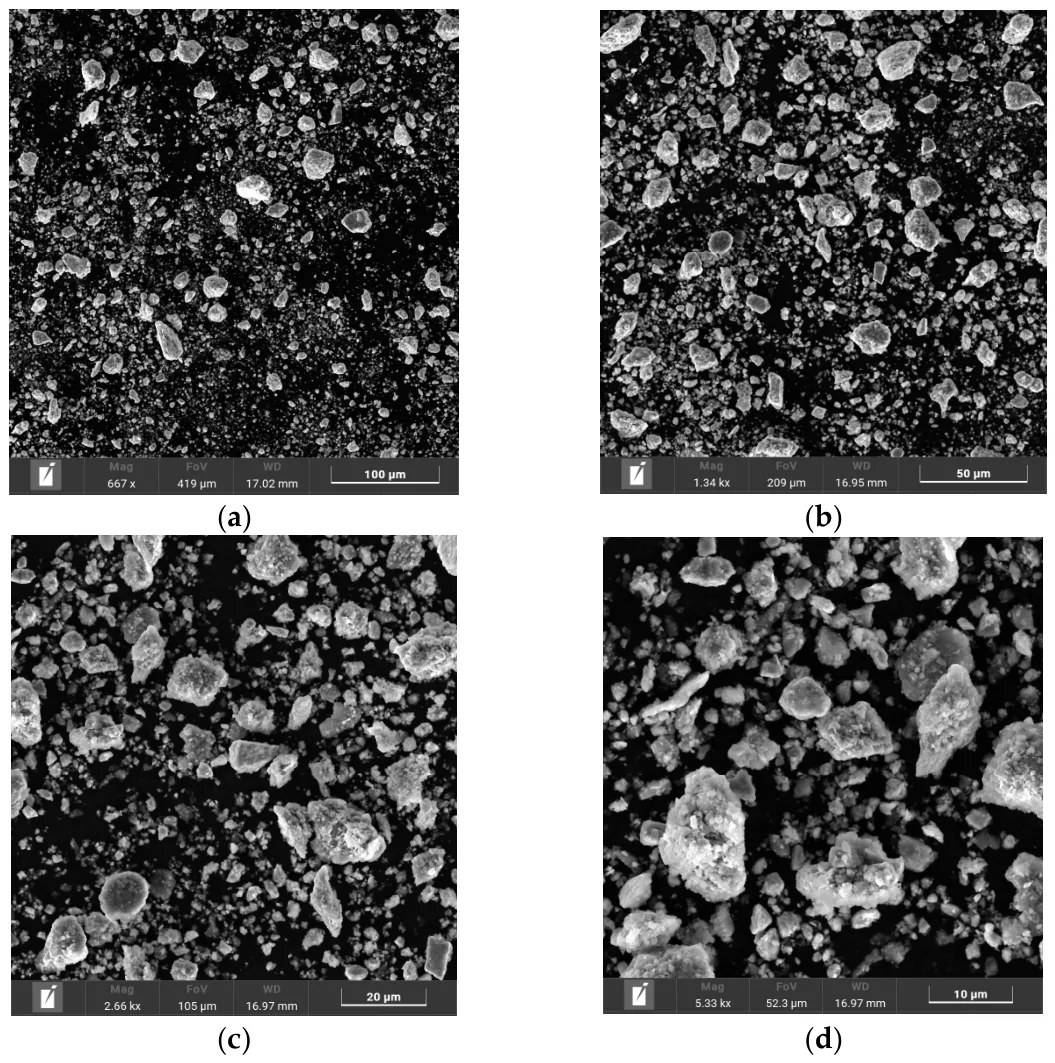
SEM images of the S 2 powder mixture after mechanical activation at 800 rpm for 40 min at different magnifications: (**a**) ×650, (**b**) ×1500, (**c**) ×2500, and (**d**) ×5000, showing the characteristic particle morphology and agglomeration of the mechanically activated powder.

Figure 11 shows the shape and morphological characteristics of the particles S 2 after mechanical activation at a planetary mill rotation speed of 800 rpm for 40 min. However, as the time is increased to 50 min, a cold welding process occurs, with powder particles adhering to the walls of the beaker and the balls. This process occurs as a result of collisions between particles and grinding balls, generating kinetic energy and causing the temperature and pressure inside the vessel to rise. In the powdered mixture under investigation, aluminum and ferrosilicon are more prone to agglomeration, as they are ductile and chemically active. Consequently, agglomeration may occur at virtually any point during mechanical activation, depending on the initial stoichiometric composition.

**Figure 12:**
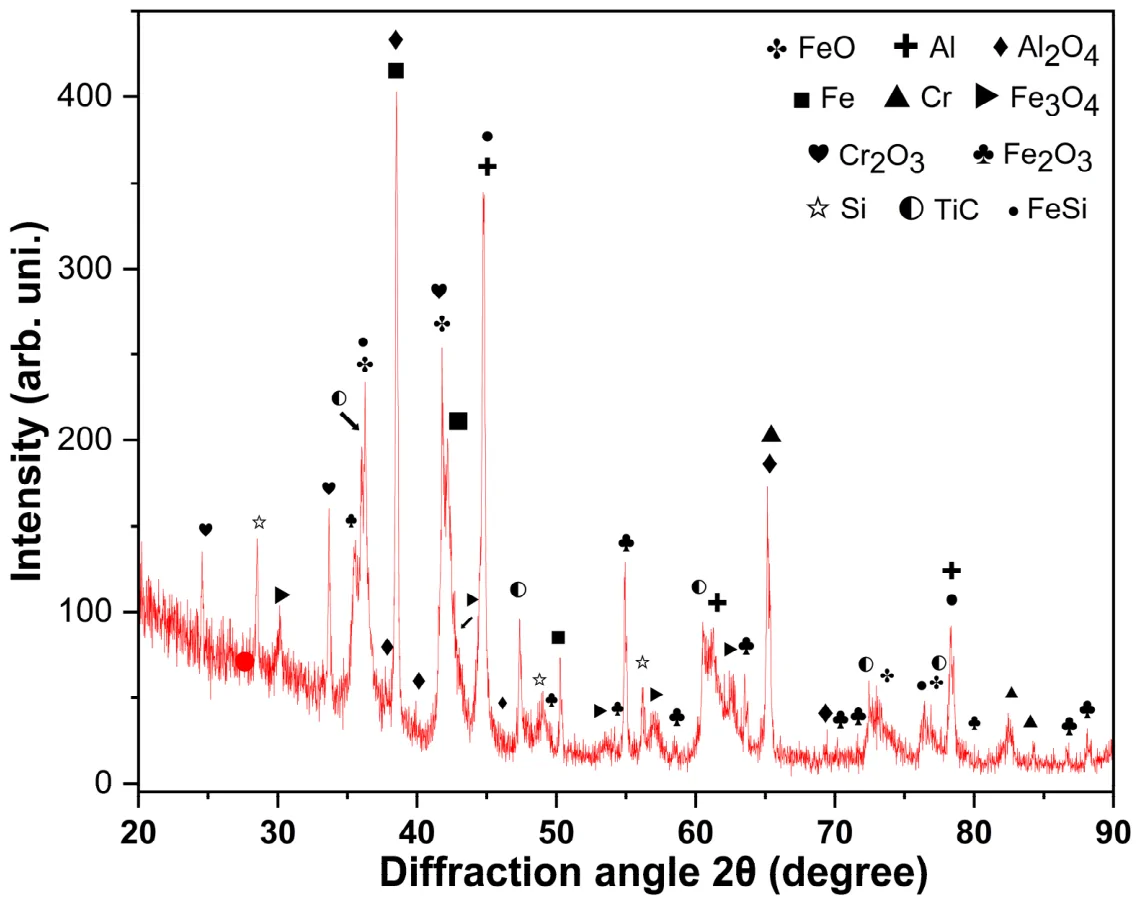
X-ray diffraction (XRD) pattern of the S 2 powder mixture after mechanical activation at 800 rpm for 40 min, showing the phase composition of the mechanically activated mixture prior to SHS.

The experiment shown in Figure 12 revealed that the diffractogram showed broadening and a decrease in the intensity of iron oxide reflections, with a reduction in iron content and an increase in aluminum content due to the aluminum powder, resulting in a shift towards higher peaks and an increase in the aluminum oxide content, as well as a decrease in the aluminum peaks. There is also a reduction of iron oxide by ferrosilicon and the formation of silicon oxide. As the chromium oxide content in the charge increases, the diffractogram shows an intense distribution of chromium oxide due to the strong interaction between the oxides and chromium and a decrease in chromium, as there is only a negligible amount of chromium present in the slag.

**Figure 13:**
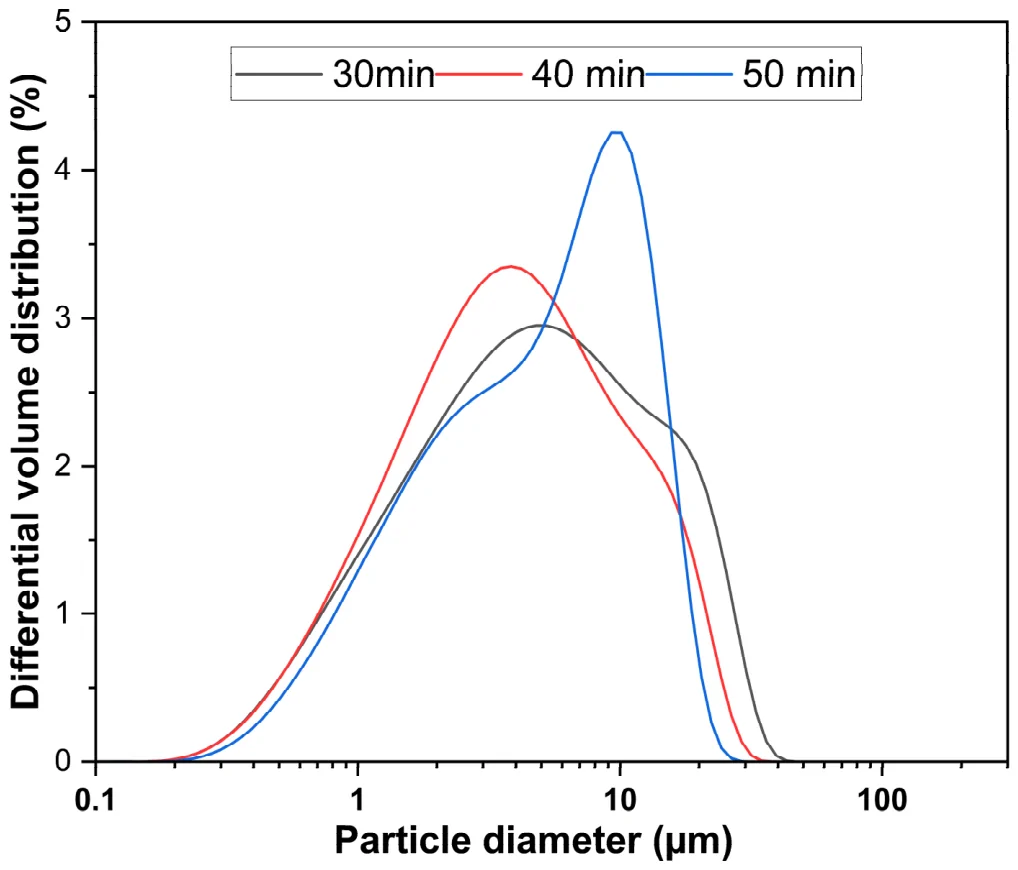
Comparison of the particle size distributions of the S 3 powder mixture after mechanical activation at 800 rpm for different activation times (30, 40, and 50 min), showing the effect of activation time on the particle size distribution.

Figure 13 and Table 7 show the particle size distribution of the powder, expressed as a percentage. The results of the analysis of the powder particles involved in the reaction show that high-energy grinding takes place, resulting in the formation of a heterogeneous dispersion with a uniform distribution of powder particles. This is due to the mechanical action on the powder particles resulting from repeated collisions and friction between the balls, the drum walls, and the powders. The grinding process resulted in the formation of a heterogeneous dispersion with a uniform distribution of components. The average particle size of the powder was 6.4 µm after 30 min, 5.5 µm after 40 min, and 3.11 µm after 50 min. This indicates that the average particle size decreased as the duration increased.

**Figure 14:**
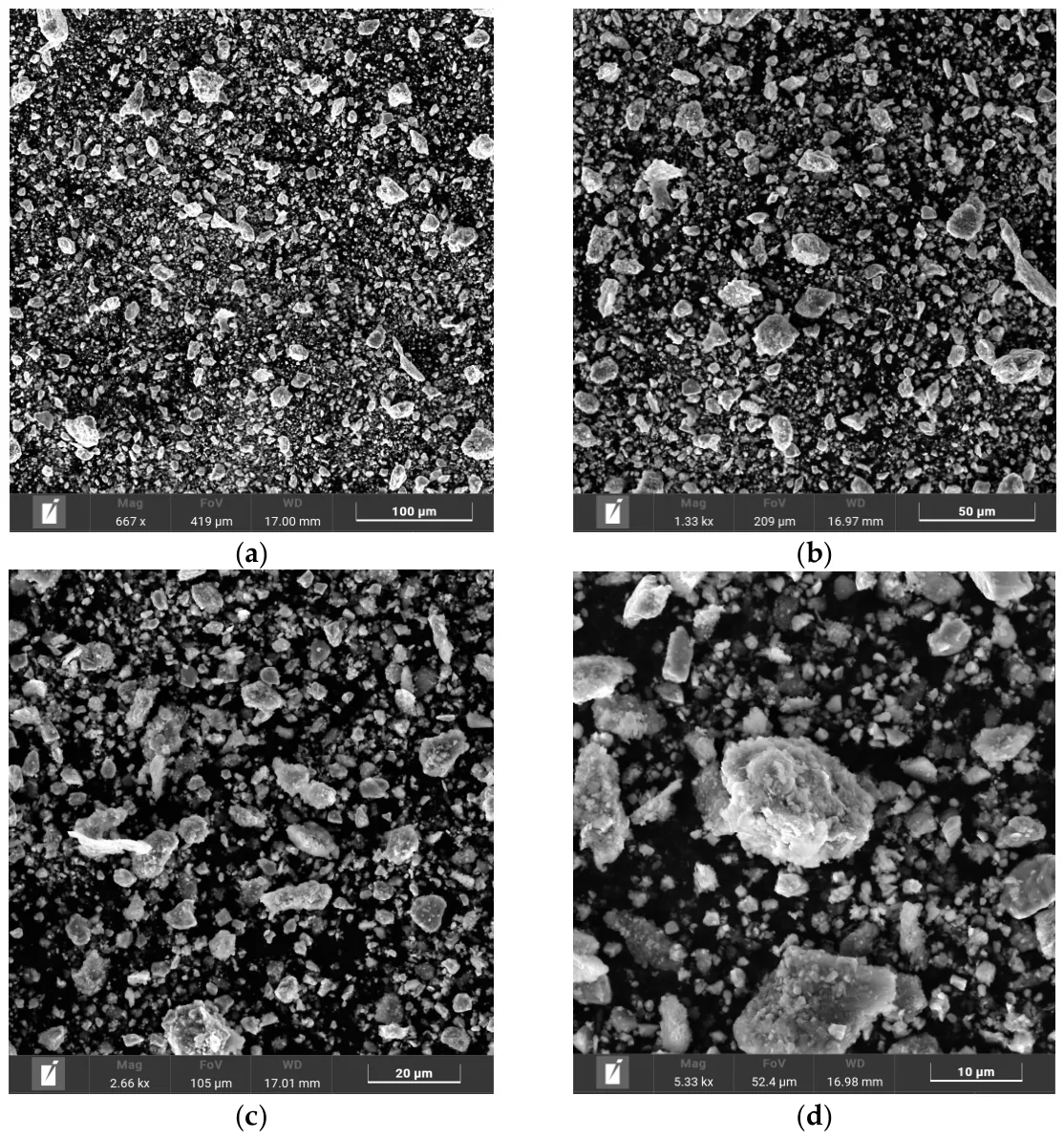
SEM images of the S 3 powder mixture after mechanical activation at 800 rpm for 40 min at different magnifications: (**a**) ×650, (**b**) ×1500, (**c**) ×2500, and (**d**) ×5000, showing the characteristic particle morphology and agglomeration of the mechanically activated powder.

Upon examining the images obtained using a scanning electron microscope (SEM) shown in Figure 14 at ×650 magnification, it becomes clear that oval-shaped particles are distributed throughout the sample. In addition, there is a small number of angular particles. At magnifications of 2500 and 5000 times, scaly particles are clearly visible. These are formed as a result of particles colliding with the grinding balls, which, in turn, is caused by high temperatures and pressures leading to melting and the formation of new phases of iron, silicon, silicon oxide, and aluminum oxide as a result of a reduction reaction.

**Figure 15:**
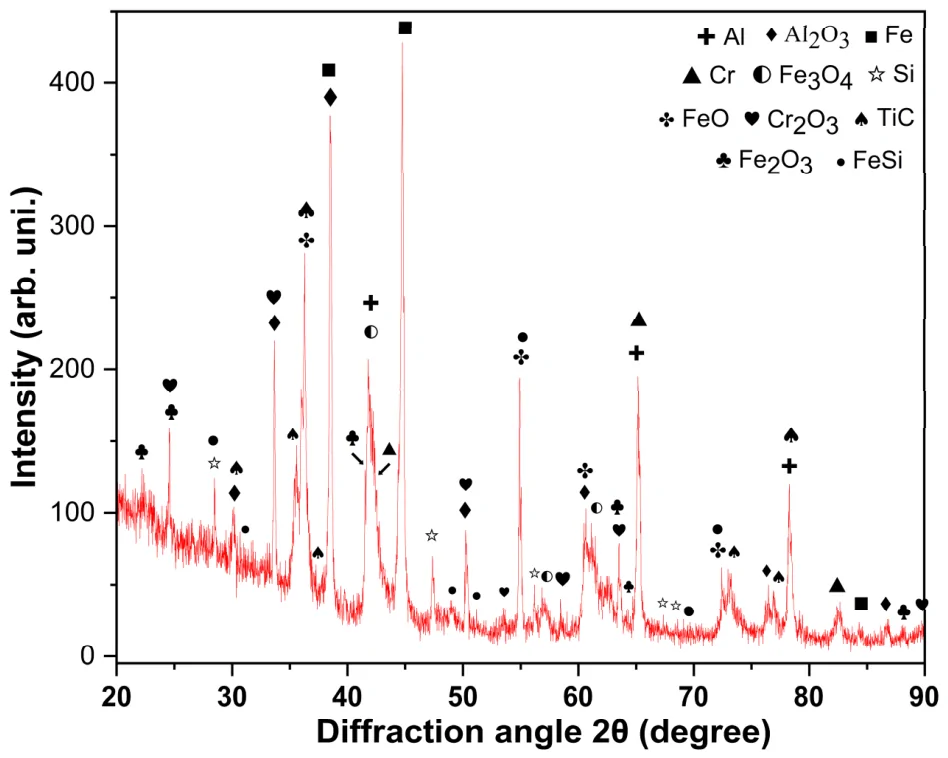
X-ray diffraction (XRD) pattern of the S 3 powder mixture after mechanical activation at 800 rpm for 40 min, showing the phase composition of the mechanically activated mixture prior to SHS.

The experiment shown in Figure 15 revealed that the diffractogram exhibited broadening and a decrease in the intensity of the iron oxide peaks, accompanied by the reduction of iron and an increase in its content under the influence of aluminum powder, as well as a shift in the peaks of aluminum oxide and ferrosilicon towards silicon and silicon dioxide. Furthermore, the crystal lattice structure changes from TiC to Ti_2_C with the formation of aluminum-chromium phases, manifesting as small peaks. All this indicates the formation of nanostructures and amorphous regions. Analysis of the X-ray diffraction patterns obtained for the powder mixture after mechanical activation (Figure 15) revealed a number of difficulties in determining the phase composition due to the overlap of the peaks of individual phases. However, alongside this, as the frequency and duration of mechanical activation increased, distinct peaks were observed for certain elements (iron, chromium, and aluminum oxides). A decrease in the maximum values relating to aluminum was also noted, which is explained by the aluminothermic reduction of iron oxide to iron. This indicates the presence of particles in the mixture that did not interact with one another during the mechanical activation process.

**Figure 16:**
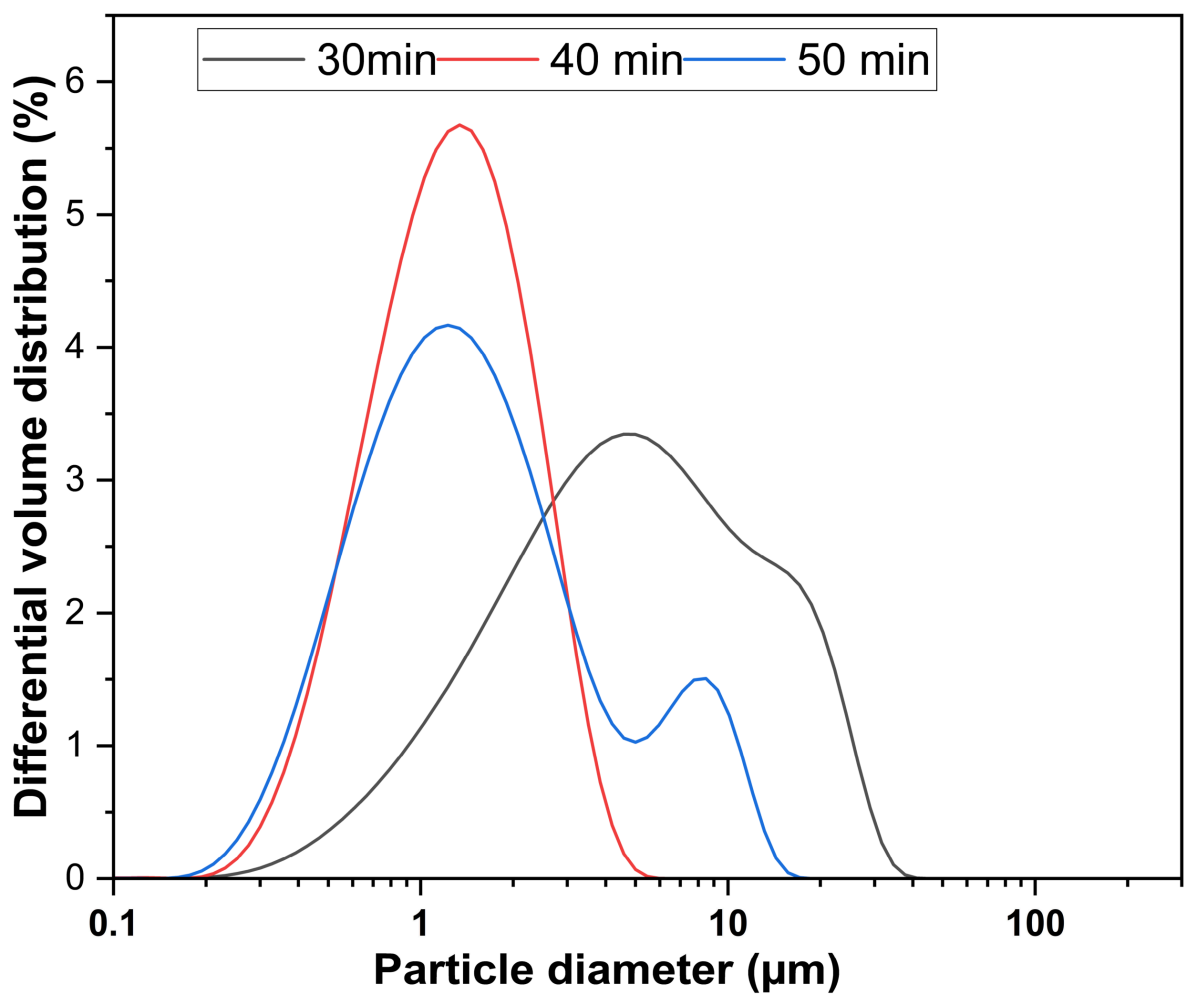
Comparison of the particle size distributions of the S 4 powder mixture after mechanical activation at 800 rpm for different activation times (30, 40, and 50 min), showing the effect of activation time on the particle size distribution.

Figure 16 and Table 8 show the particle size distribution of the powder, expressed as a percentage. The results obtained from the particle analyzer tests show that high-energy grinding takes place, resulting in the formation of a heterogeneous, dispersed mixture with a uniform distribution of powder particles. This is due to the mechanical action on the powder particles resulting from repeated collisions and friction between the balls, the drum walls, and the powders. As a result of the grinding, a heterogeneous dispersed mixture with a uniform distribution of components was observed. The average particle size of the powder was 6.9 µm after 30 min, 4.2 µm after 40 min, and 2.4 µm after 50 min. This indicates that increasing the grinding time leads to a decrease in the average particle size.

**Figure 17:**
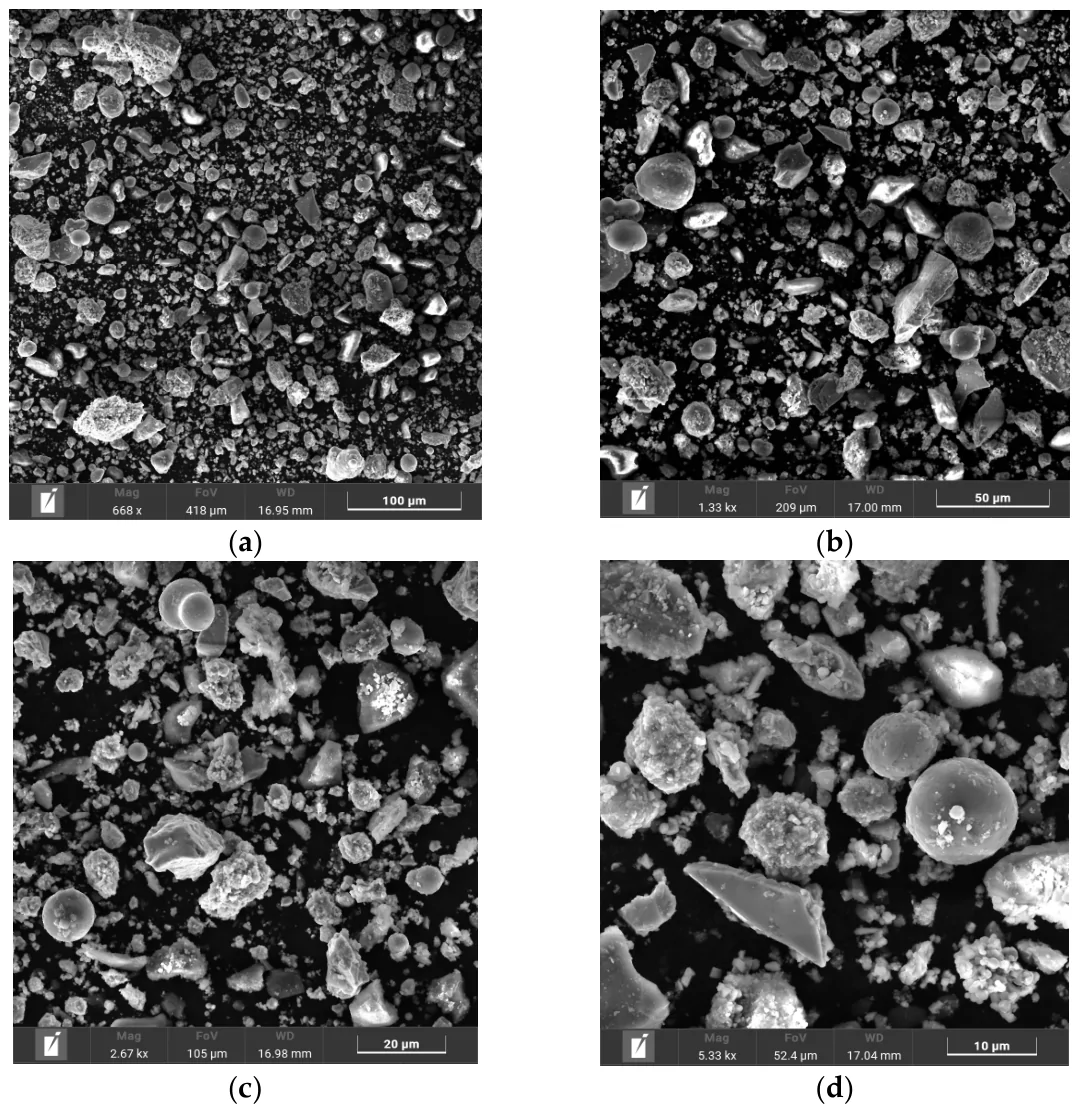
Comparison of SEM images of the S 4 powder mixture after mechanical activation at 800 rpm for 40 min at different magnifications: (**a**) ×650, (**b**) ×1500, (**c**) ×2500, and (**d**) ×5000, showing the morphology and particle-size distribution of the mechanically activated powder.

Figure 17 shows the particle morphology of the powders, as observed using a scanning electron microscope (SEM); the images reveal that the powders have rounded, needle-like, and flake-like morphologies, which ensure a denser and more uniform distribution of particles within the powder, thereby having a beneficial effect on the subsequent sintering process.

**Figure 18:**
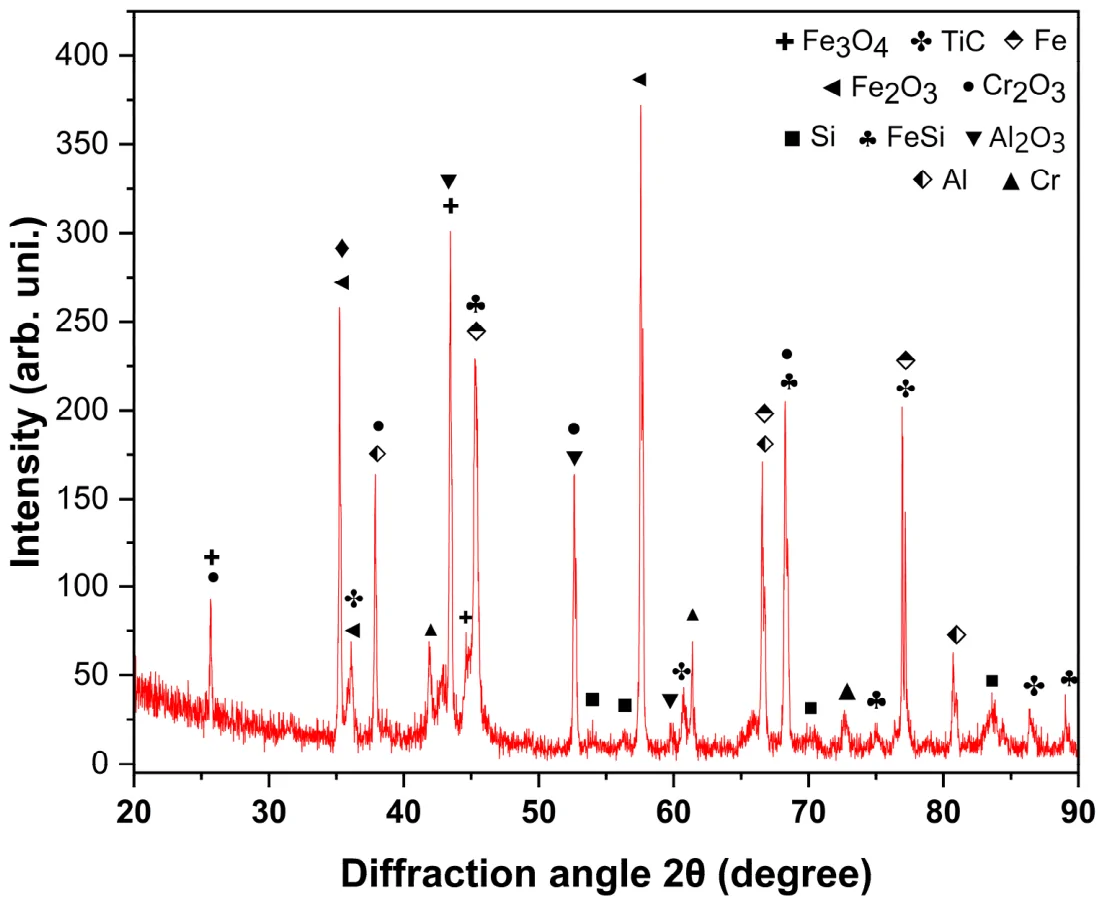
X-ray diffraction (XRD) pattern of the S 4 powder mixture after mechanical activation at 800 rpm for 40 min, showing the phase composition of the mechanically activated mixture prior to SHS.

Figure 18 presents an analysis of the X-ray diffraction peaks obtained for the powder mixture following mechanical activation; this analysis revealed a number of difficulties in determining the phase composition due to the overlap of most of the strong lines from the identifiable phases. However, alongside this, during mechanization lasting up to 30 min at a rotation speed of 800 rpm for all powder mixtures with different particle sizes, high peaks of individual elements were observed: iron oxides, chromium oxide, and aluminum. A decrease in the aluminum peaks was also observed; this is attributed to the aluminothermic reduction of iron oxide (hematite) to iron. At a holding time of 40 min, there is also no change in phase transformations, but the difference is that small peaks of aluminum oxide, low in intensity, appear. This indicates the presence of particles in the mixture that did not interact with one another during the mechanical processing. It follows that, due to the high rotational speeds inside the beaker and the small size of the grinding beads (5 mm in diameter), as the processing time increases to 50 min, the plastic deformation of the particles intensifies and agglomeration of the powder particles on the walls occurs.

### 3.3. Synthesis of the Reaction by the Self-Propagating High-Temperature Synthesis Method

When carrying out the sintering reaction of powder charge compositions comprising scale from alloy steel, aluminum, aluminum oxide, chromium oxide, titanium carbide, and ferrosilicon using the SHS method in air, an inert (argon) atmosphere, or a vacuum. The ignition of the mixture of thermite and iron oxide resulted in the release of a large amount of heat. The heat released caused the charge to burn, transferring its energy via heat transfer from the base to the layer in a frontal combustion regime. As the powder mixture burned, it melted, forming a new composite material structure through the diffusion of chemical elements into the newly formed material. The sintering process resulted in the formation of a metal-ceramic material characterized by a rigid framework and a porous structure, which arises due to the redistribution of the melt within the reaction zone and the desorption of gases. As a consequence of these processes, the pores have an irregular shape and can be either through-pores (predominantly) or blind pores. Through-pores have a tortuous structure with a rough surface.

**Figure 19:**
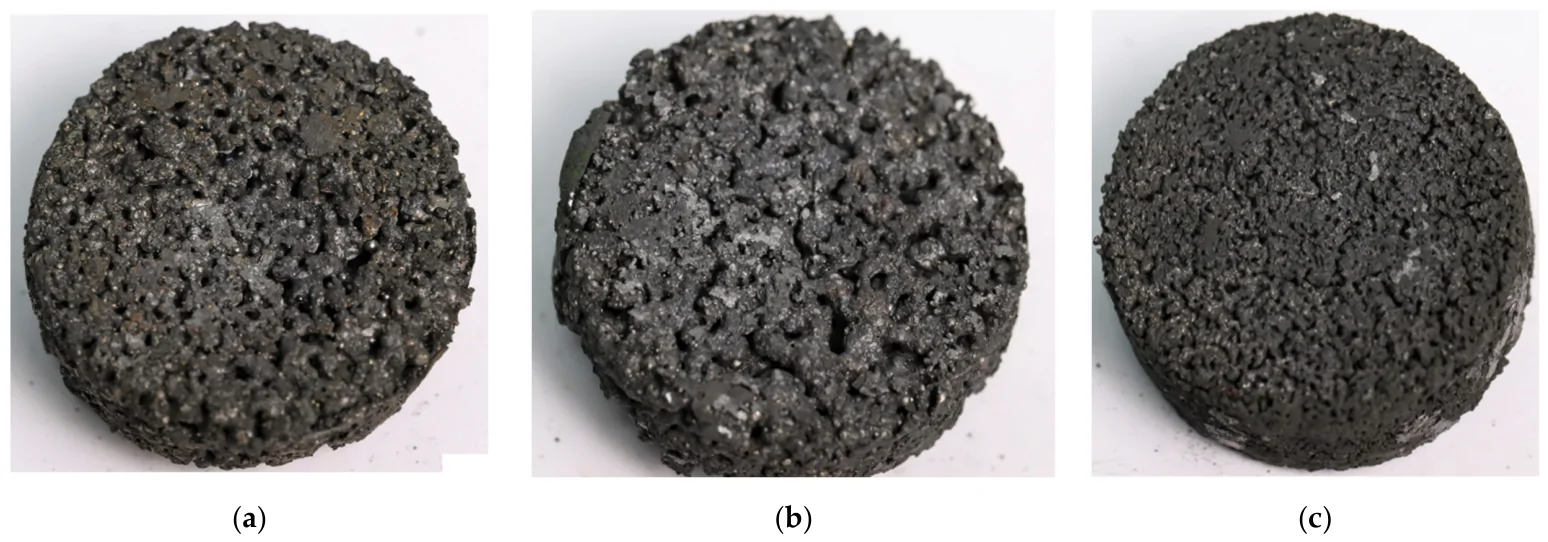
Comparison of the macroscopic appearance of SHS products synthesized from the S 4 powder mixture (FeO-45 wt.%, Al-20 wt.%, Al_2_O_3_-7.5 wt.%, Cr_2_O_3_-5 wt.%, TiC-10 wt.%, and FeSi-12.5 wt.%) under different atmospheres: (**a**) argon, (**b**) air, and (**c**) vacuum, showing the effect of the synthesis atmosphere on the macroscopic structure and integrity of the resulting porous products.

Figure 19 shows microstructural images of samples obtained following synthesis in various environments. Analysis of the microstructure reveals that, when synthesis is carried out in a vacuum, the average pore diameter is significantly smaller compared to samples obtained in air and inert atmospheres. At the same time, an increase in the number of pores and a more uniform distribution of these pores throughout the material’s volume is observed.

**Figure 20:**
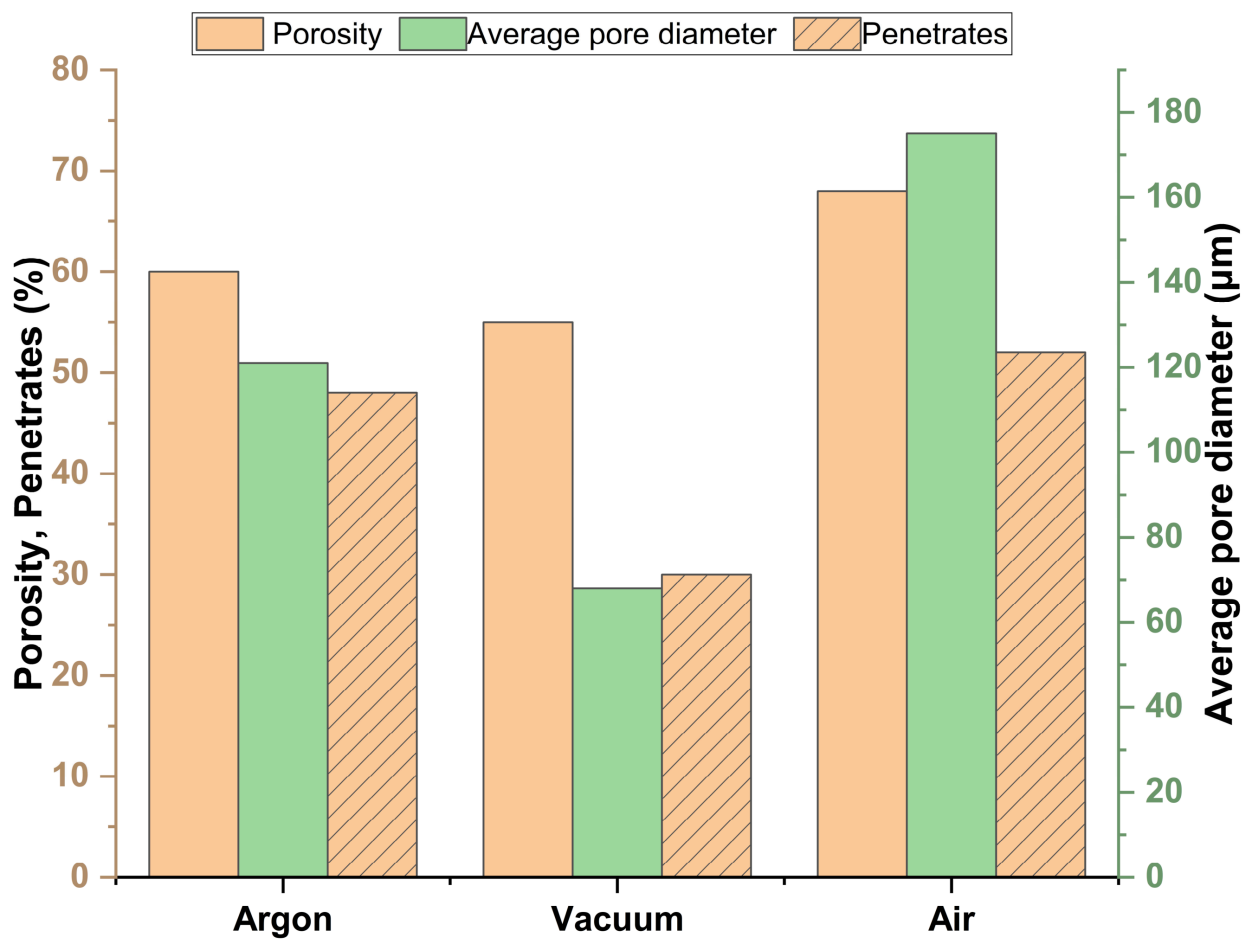
Comparison of the porosity, permeability, and average pore diameter of the S 4 porous cermet synthesized by self-propagating high-temperature synthesis (SHS) under different atmospheres (air, argon, and vacuum), showing the effect of the synthesis atmosphere on the resulting pore structure.

According to the analysis results presented in Figure 20, the porosity of the samples obtained in an argon atmosphere was 60%, and the permeability was 48%. For samples synthesized in a vacuum, the porosity and permeability were 55% and 30%, respectively. The highest values were recorded for samples obtained in an air atmosphere: the porosity was 68%, and the permeability was 52%. The formation of a fine-pored structure in a vacuum is due to intensive degassing associated with the presence of oxide components in the feedstock, as well as low-pressure conditions under which gaseous reaction products are effectively removed before pore coarsening begins. This results in a structure with a high density of fine pores. Analysis of the results presented in Figure 20 showed that the average pore diameter during synthesis in an air atmosphere was 175 µm, in an argon atmosphere was 121 µm, and in a vacuum was 68 µm. The increase in the average pore size during synthesis in air is associated with the occurrence of additional exothermic reactions involving the oxidation of aluminum and the metallic components of the charge by oxygen.

**Figure 21:**
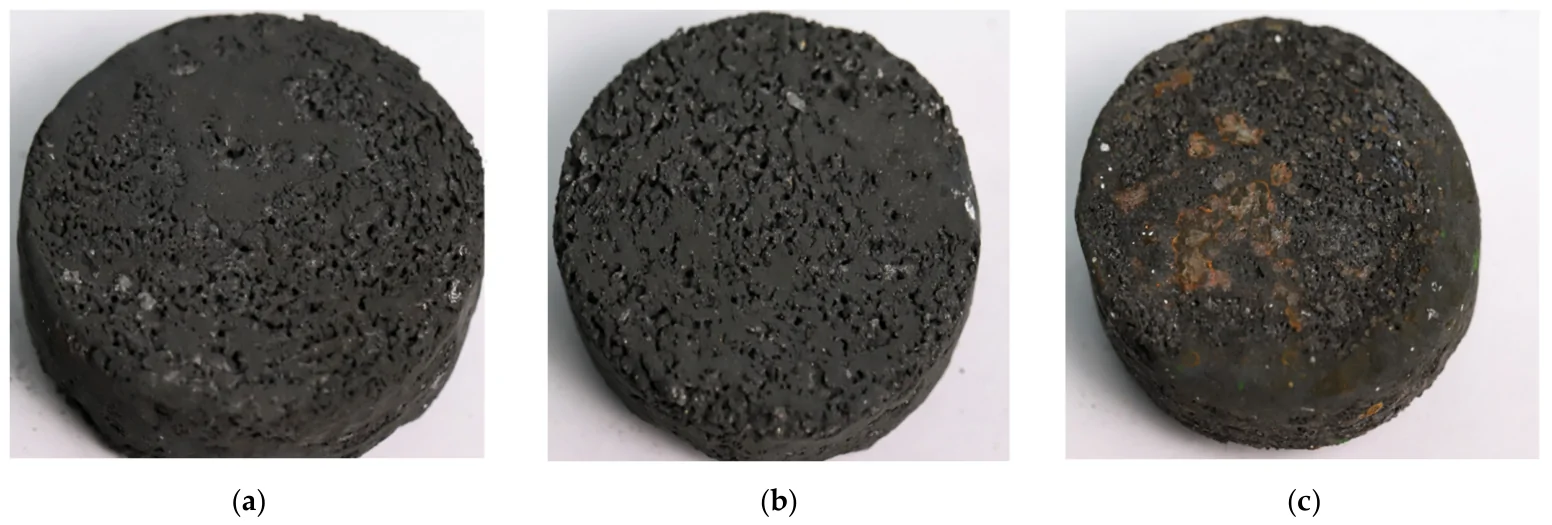
Comparison of the macroscopic appearance of SHS products synthesized from the S 3 powder mixture (FeO-40 wt.%, Al-20 wt.%, Al_2_O_3_-10 wt.%, Cr_2_O_3_-5 wt.%, TiC-12.5 wt.%, and FeSi-12.5 wt.%) under different atmospheres: (**a**) argon, (**b**) air, and (**c**) vacuum, showing the effect of the synthesis atmosphere on the macroscopic structure and integrity of the resulting porous products.

**Figure 22:**
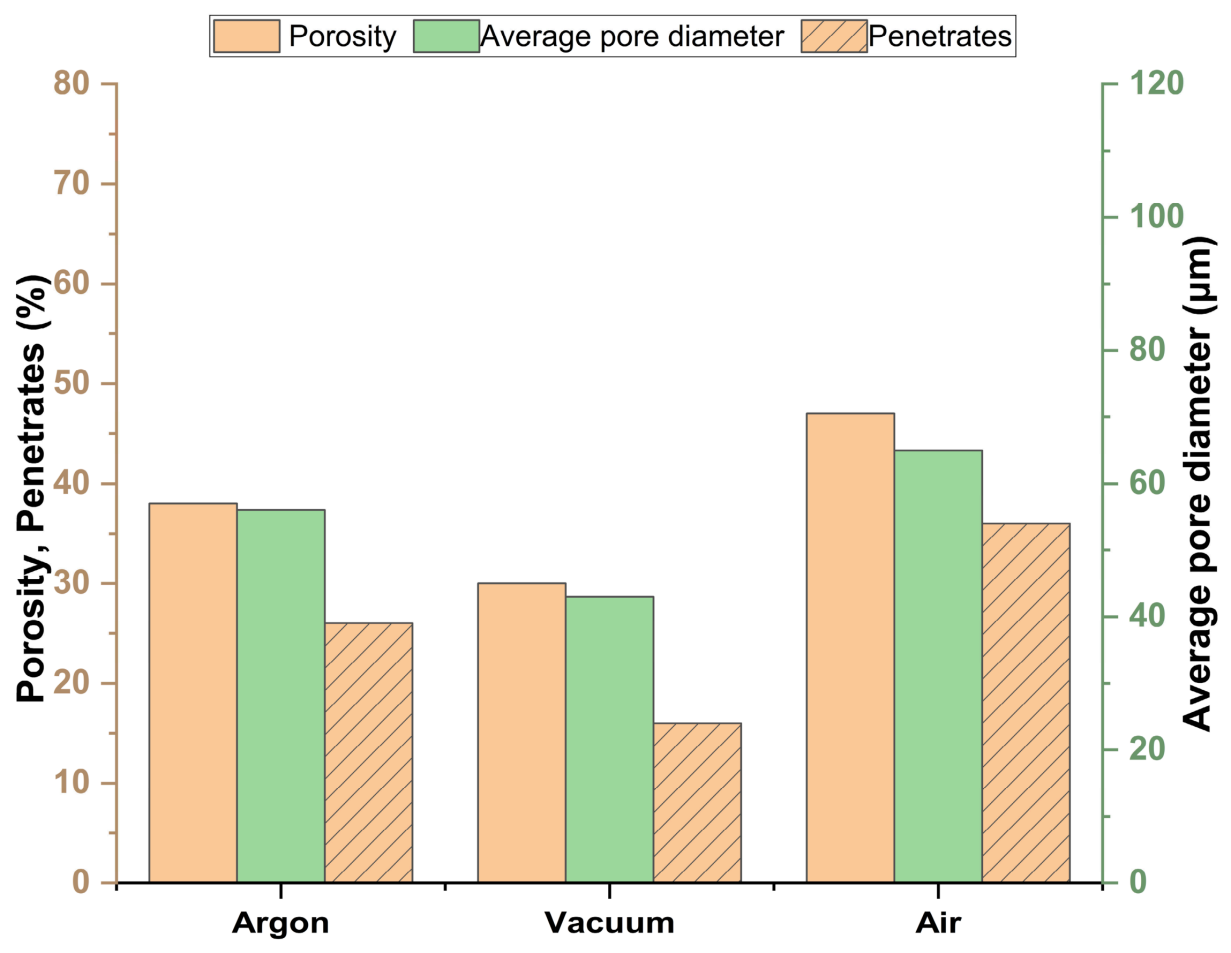
Comparison of the porosity, permeability, and average pore diameter of the S 3 porous cermet synthesized by self-propagating high-temperature synthesis (SHS) under different atmospheres (air, argon, and vacuum), showing the effect of the synthesis atmosphere on the resulting pore structure.

Figure 21 shows the microstructure of samples obtained from mixture 3 using the self-propagating high-temperature synthesis (SHS) method. The figure clearly shows that when synthesis is carried out in a vacuum, there is a reduction in the average pore diameter and total porosity compared to samples obtained in air and inert atmospheres. A comparative analysis of samples synthesized in air and in an inert atmosphere showed that the average pore diameter in air is higher than in an inert atmosphere. At the same time, the number of pores in the samples under investigation does not change significantly, as confirmed by the results shown in Figure 22.

Figure 22 shows the results of the determination of the porosity, permeability, and average pore diameter of the samples obtained. It was found that during synthesis in an air atmosphere, porosity and permeability were 47% and 36%, respectively; in an argon atmosphere, 38% and 26%; and under vacuum conditions, 30% and 16%. The average pore diameter for samples obtained in air is 65 µm, in an inert atmosphere (argon) is 56 µm, and in a vacuum is 43 µm.

**Figure 23:**
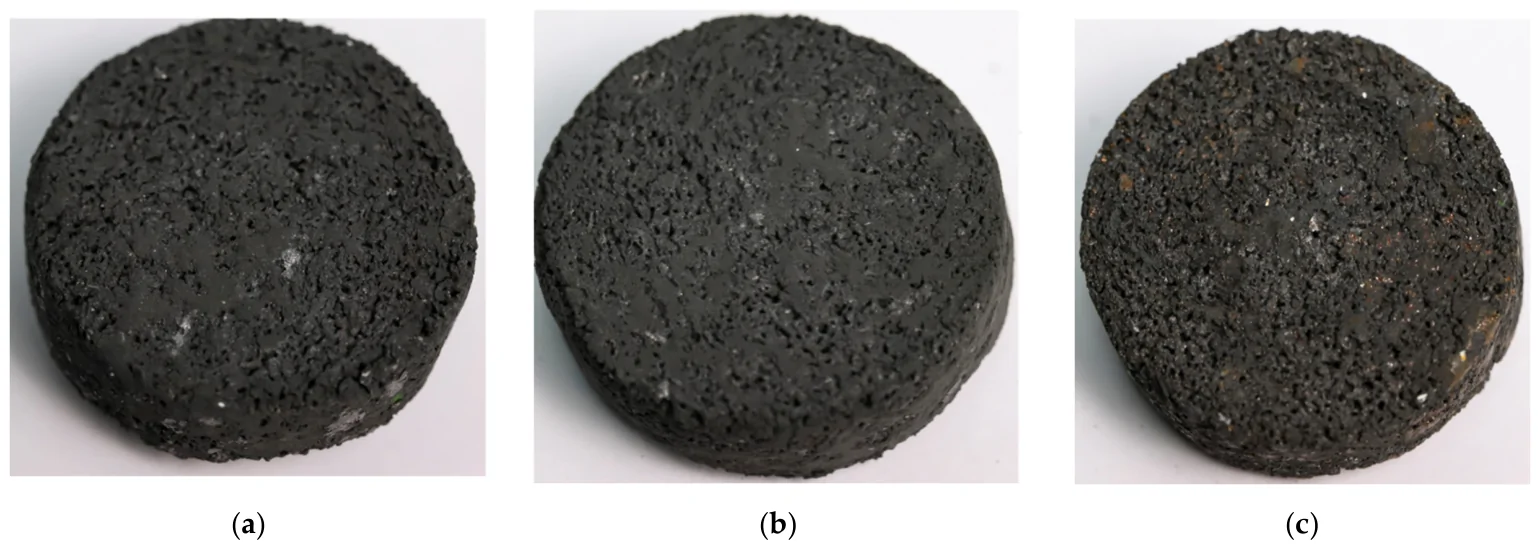
Comparison of the macroscopic appearance of SHS products synthesized from the S 2 powder mixture (FeO-40 wt.%, Al-20 wt.%, Al_2_O_3_-7.5 wt.%, Cr_2_O_3_-7.5 wt.%, TiC-12.5 wt.%, and FeSi-12.5 wt.%) under different atmospheres: (**a**) argon, (**b**) air, and (**c**) vacuum, showing the effect of the synthesis atmosphere on the macroscopic structure and integrity of the resulting porous products.

When carrying out the reaction using the self-propagating high-temperature synthesis (SHS) method for a charge composition of FeO-40%, Al-20%, Al_2_O_3_-7.5%, Cr_2_O_3_-7.5%, TiC-12.5%, and FeSi-12.5%, it was found that synthesis in ambient air leads to a reduction in both the number of pores and their average diameter compared with samples obtained in an inert atmosphere. These results are presented in Figure 23. For samples synthesized in an inert atmosphere, a reduction in both total porosity and the average pore diameter is also observed. The same phenomenon is observed in a vacuum environment. This effect may be associated with slowed heat removal in the ambient, inert, and vacuum environments, which contributes to an increase in the liquid phase and a reduction in the release of gaseous reaction products. As a result, a more developed porous structure with reduced pore sizes is formed.

**Figure 24:**
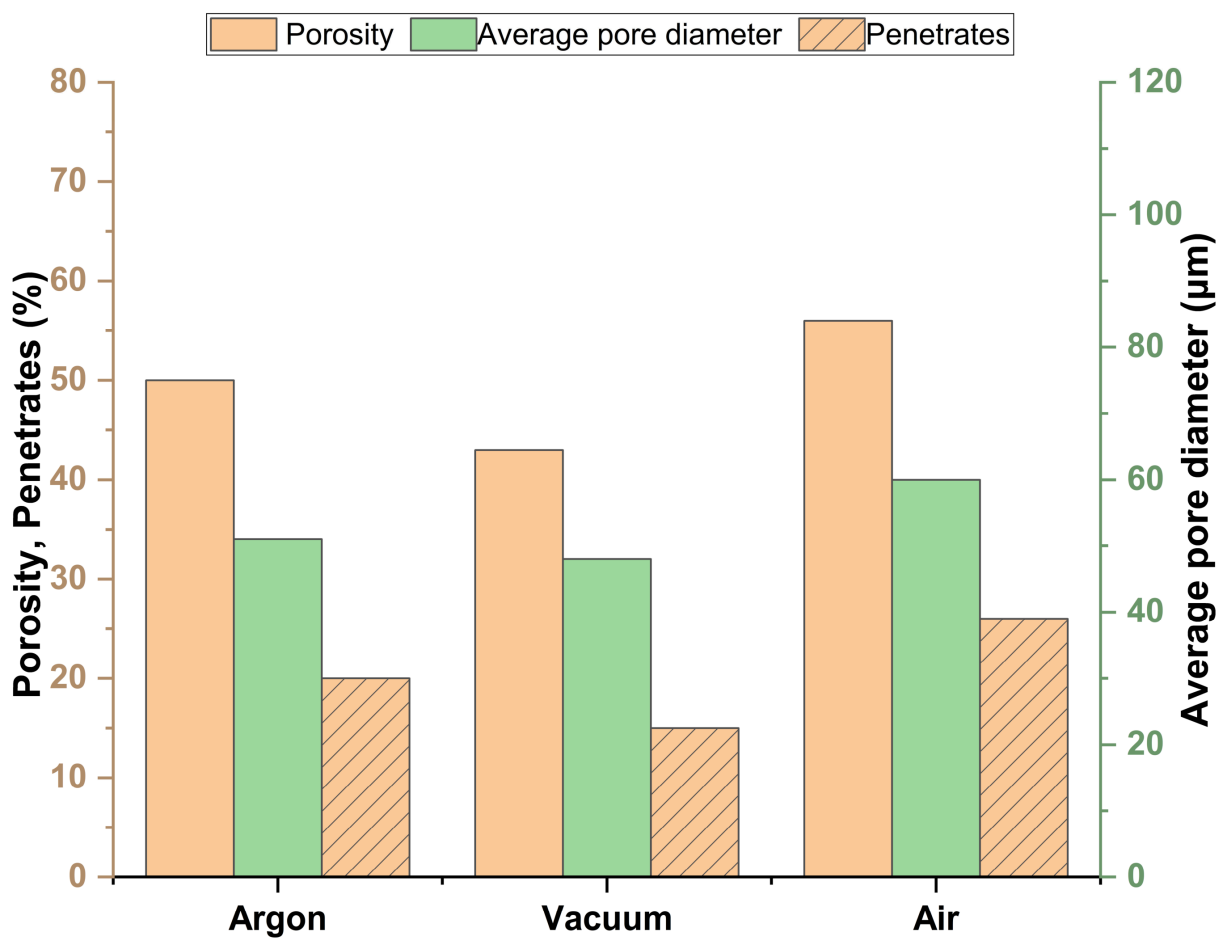
Comparison of the porosity, permeability, and average pore diameter of the S 2 porous cermet synthesized by self-propagating high-temperature synthesis (SHS) under different atmospheres (air, argon, and vacuum), showing the effect of the synthesis atmosphere on the pore structure and permeability of the resulting material.

Figure 24 shows the results of the determination of the porosity, permeability, and average pore diameter of the samples obtained. It was found that during synthesis in air, the porosity and permeability were 56% and 26%, respectively; in an argon atmosphere, 50% and 20%; and under vacuum conditions, 43% and 15%. The average pore diameter for samples obtained in air is 60 µm, in an inert atmosphere (argon) is 51 µm, and in a vacuum is 48 µm.

By comparing Figure 21, Figure 22, Figure 23 and Figure 24 of the microstructural analysis, the aim was to identify the influence of the chemical composition of the charge, in particular aluminum oxide and chromium oxide, on the pore formation processes. The results showed that when the scale of alloyed steel reacts with aluminum, an intense aluminothermic reaction occurs, accompanied by the intense release of heat and gaseous products. As a result of phase transformation during the transition from the liquid to the solid state, gas voids form pores within the material’s structure. When aluminum oxide is added to the charge, it does not, as it were, participate in the aluminothermic process but acts as a thermal regulator, thereby reducing the combustion temperature and intensity, thus serving to regulate phase formation and the structure of the material.

This is confirmed by Figure 21 and Figure 22: as the proportion of aluminum oxide increases and that of chromium oxide decreases, the intensity of combustion and heat release decreases; the liquid phase fills the voids as gases are released, thereby reducing the number of pores. A comparison of Figure 21, Figure 22, Figure 23 and Figure 24 shows that the identified patterns remain consistent across sintering conditions, which confirms the reliability of the experimental results obtained.

Furthermore, it can be stated that as the proportion of chromium oxide increases, the average pore diameter increases, as clearly shown in Figure 23. This is confirmed by the chemical reaction: the reduction of chromium oxide with additional heat release, an increase in the volume of the liquid phase, and intensified gas formation, which leads to an increase in pore size within the structure of the metal-ceramic material:

Cr_2_O_3_ + 2Al → 2Cr + Al_2_O_3_ + Q, (12)

**Figure 25:**
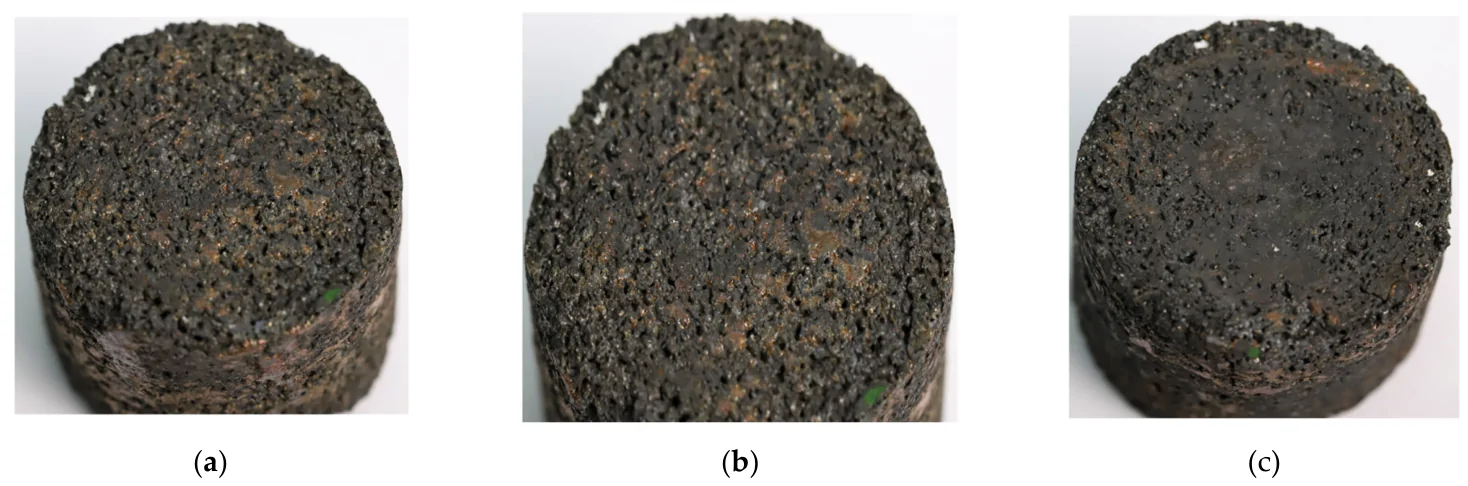
Comparison of the macroscopic appearance of SHS products synthesized from the S 1 powder mixture (FeO-40 wt.%, Al-20 wt.%, Al_2_O_3_-7.5 wt.%, Cr_2_O_3_-5 wt.%, TiC-10 wt.%, and FeSi-17.5 wt.%) under different atmospheres: (**a**) argon, (**b**) air, and (**c**) vacuum, showing the effect of the synthesis atmosphere on the macroscopic structure and integrity of the resulting porous products.

Figure 25 illustrates the process of self-propagating high-temperature synthesis carried out under vacuum conditions; the porosity and pore diameter decreased compared with sintering in an inert atmosphere. In this case, the porosity was distributed throughout the entire volume, and the number of pores increased compared to the vacuum mode, whilst the average pore diameter decreased; however, the porosity remained unchanged compared to the ambient atmosphere mode, but the average pore diameter was greater than in the argon and vacuum modes. This confirms that during the sintering process, under the influence of the sintering atmosphere, oxide compounds actively interact with the air during combustion, oxidizing and thereby regulating the formation of macro- and micropores and their number, thus influencing the structure of the material. Furthermore, increasing the proportion of ferrosilicon in the charge to 17.5% leads, upon combustion of the charge with the formation of a liquid phase, to the filling of pore voids and a reduction in the melting temperature, resulting in the formation of a liquid, fluid solution accompanied by the reaction:

FeSi → Fe + Si,(13)

The material structure is also influenced by a reduction in the amount of aluminum oxide. As a result, a dense material structure is formed with a reduced pore volume; this is achieved by filling the structure with a fluid solution under vacuum, leading to the formation of a dense structure and a reduction in pore size. In an inert atmosphere, the reduced silicon is distributed throughout the metal-ceramic framework with the released iron, further strengthening it with iron, as argon is a neutral gas that does not react with other metals. During sintering in air, silicon and iron partially react with the air, oxidizing; the temperature rises, the pore size increases, and the material is distributed throughout the entire metal-ceramic structure.

**Figure 26:**
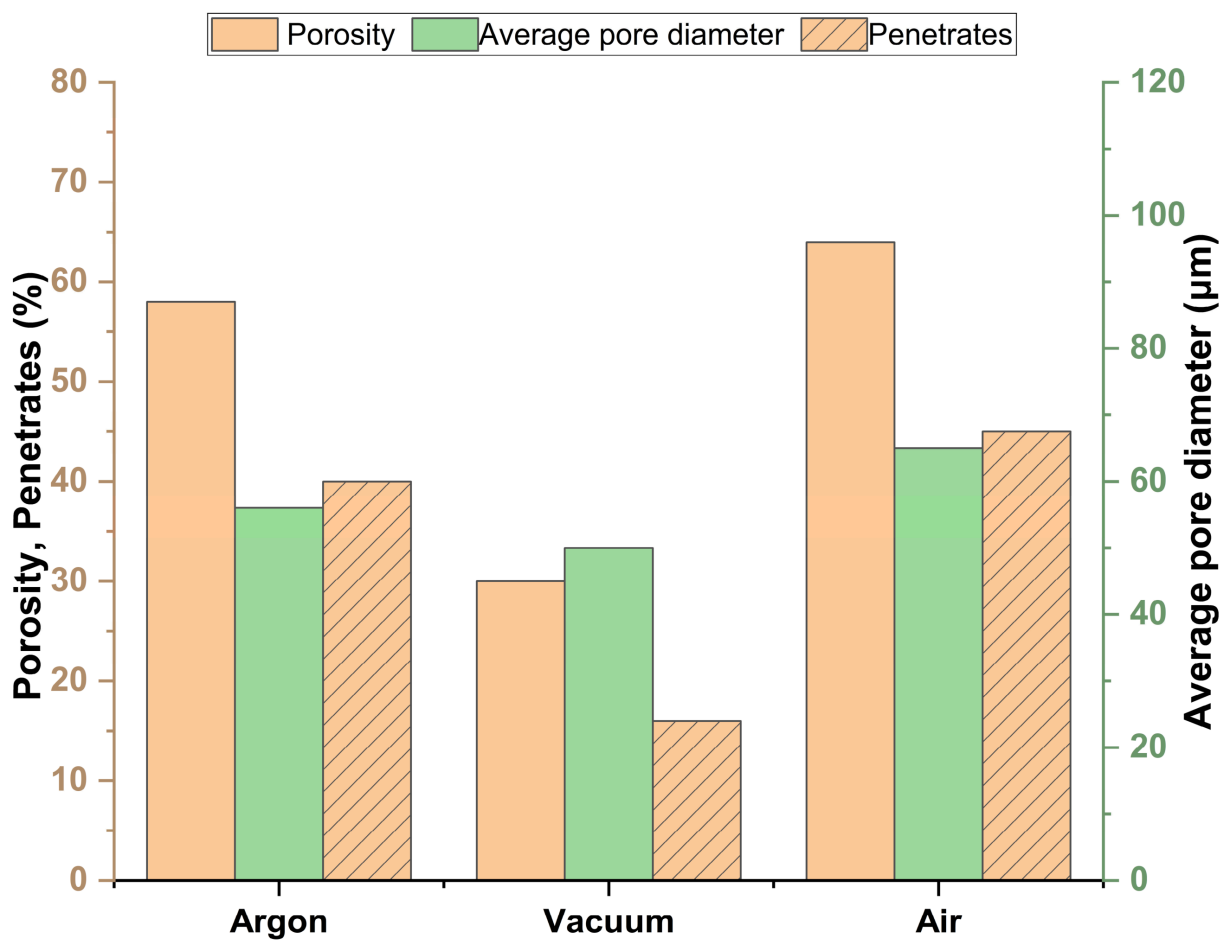
Comparison of the porosity, permeability, and average pore diameter of the S 1 porous cermet synthesized by self-propagating high-temperature synthesis (SHS) under different atmospheres (air, argon, and vacuum), showing the effect of the synthesis atmosphere on the pore structure and gas-transport characteristics.

Figure 26 shows the results of the determination of the porosity, permeability, and average pore diameter of the samples obtained. It was found that during synthesis in air, the porosity and permeability were 64% and 45%, respectively; in an argon atmosphere, 58% and 40%; and under vacuum conditions, 30% and 16%. The average pore diameter for samples obtained in air is 65 µm, in an inert atmosphere (argon) is 56 µm, and in a vacuum is 50 µm.

It follows that, through chemical interaction—specifically, as the slag content increases—there is an intense reaction with aluminum, releasing heat; however, the temperature drop that results in the release of gases is controlled by the porosity of the aluminum oxide. In addition, we use ferrosilicon to lower the temperature by distributing heat throughout the entire volume, thereby regulating the number of pores through the uniform distribution of the liquid phase. The average pore diameter decreases with the introduction of chromium oxide; as the chromium oxide content in the charge increases, the average pore diameter increases. Furthermore, the average pore diameter is influenced by the sintering atmosphere; in air, oxygen additionally reacts during combustion. In a vacuum environment with low air content, the average pore diameter decreases. In an inert atmosphere, the combustion temperature is high, but no additional air interacts, meaning there is no combustion reaction. The sintering atmosphere also affects the porosity of the material; the vacuum atmosphere is the optimal choice in all cases. This is because, with a minimal amount of air, interaction with the chemical elements is minimal, and the chemical composition of ferrosilicon is additionally influenced.

Based on the results of the study of the microstructure, porosity, permeability, and average pore size of samples obtained using different chemical compositions of the feedstock and under various synthesis conditions, it was established that feedstock No. 4 (S-4) exhibits the most optimal characteristics. Compared with the other compositions studied, this sample is characterized by higher values of porosity and permeability, as well as an increased average pore size.

The results obtained indicate that changes in the chemical composition of the feedstock and in the conditions of self-propagating high-temperature synthesis have a significant influence on the formation of the material’s porous structure. It has been established that the use of charge No. 4 (S-4) allows for the formation of the most developed porous structure with an optimal combination of porosity, permeability, and mean pore size, making this composition the most promising for the production of porous materials with improved performance characteristics.

**Figure 27:**
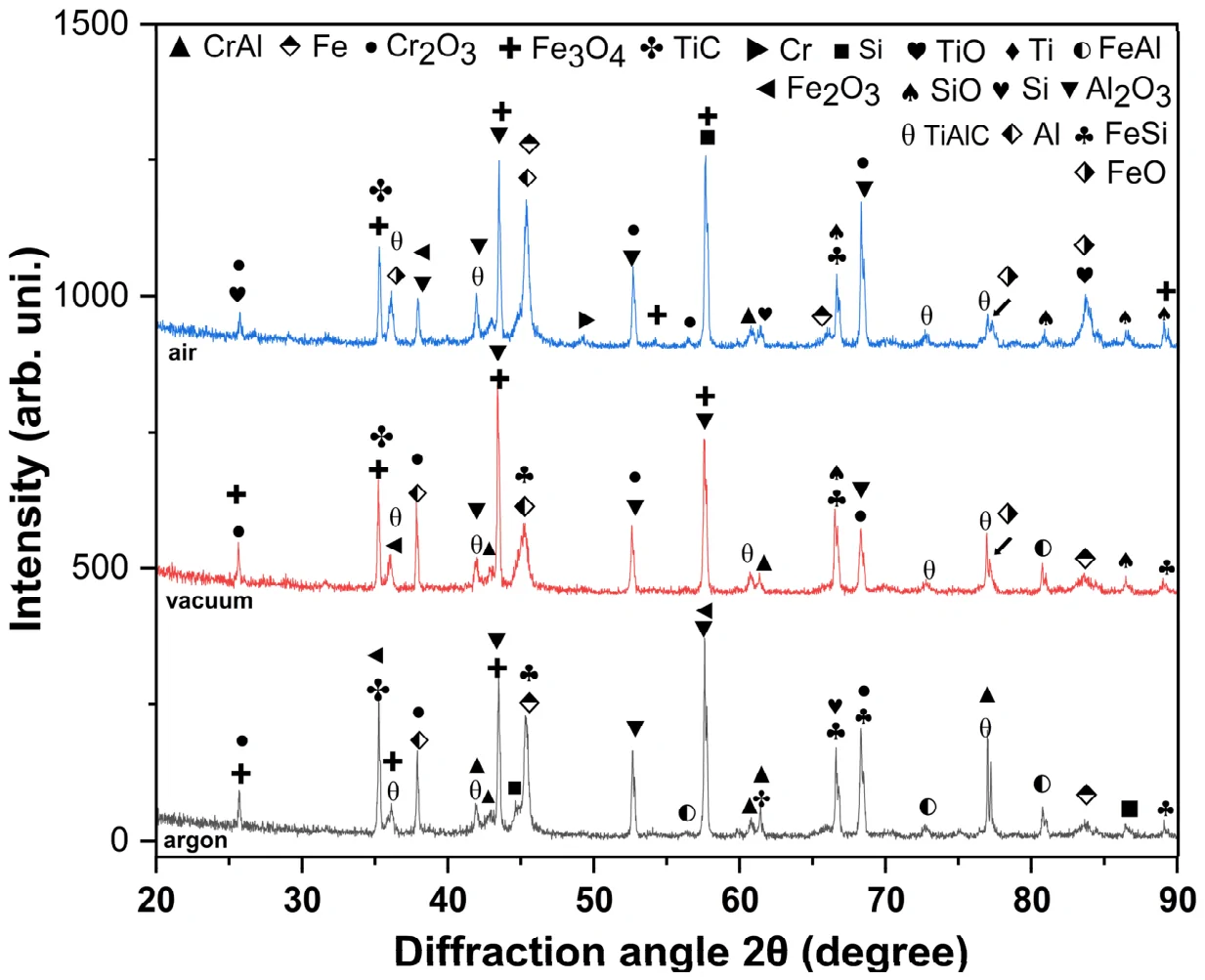
Comparison of X-ray diffraction (XRD) patterns for the S 4 mixture synthesized by SHS under different atmospheres: vacuum, air, and argon, showing phase evolution.

The X-ray diffraction patterns shown in Figure 27 compare the phase compositions of the SHS products synthesized under argon, vacuum, and air atmospheres. Under vacuum conditions, the diffraction peak intensities associated with iron, chromium, and aluminum oxides decreased, whereas those corresponding to Fe–Al and Cr–Al intermetallic phases became more pronounced. These changes may be attributed to the low oxygen partial pressure, which can promote the reduction or decomposition of thermodynamically unstable oxide phases and favor the formation of metallic and intermetallic constituents. Changes in the FeSi diffraction peaks were also observed under oxygen-deficient conditions, suggesting differences in phase evolution during SHS. These conditions may promote the formation of a transient low-viscosity liquid phase, which could facilitate particle rearrangement and interparticle bonding through capillary forces, thereby contributing to structural densification and the observed reduction in porosity and average pore diameter. In contrast, the samples synthesized in air exhibited more pronounced diffraction peaks associated with iron, chromium, and aluminum oxides and lower relative intensities of FeSi, TiC, Ti–Al–C, and intermetallic-phase peaks. The enhanced oxidation under air may restrict densification and promote the development of a more open porous structure, consistent with the experimentally observed increase in total porosity and average pore diameter.

During SHS in air, the formation of the material structure occurs under oxygen-rich conditions, which favor the formation and stabilization of refractory oxide phases. This may suppress reactions leading to the formation of intermetallic and carbide-containing phases, consistent with the lower relative intensities of their diffraction peaks. Enhanced oxidation may also affect pore evolution during SHS, promoting pore growth and coalescence and consequently increasing the average pore diameter and total porosity. These structural changes may adversely affect the mechanical properties of the synthesized material.

**Figure 28:**
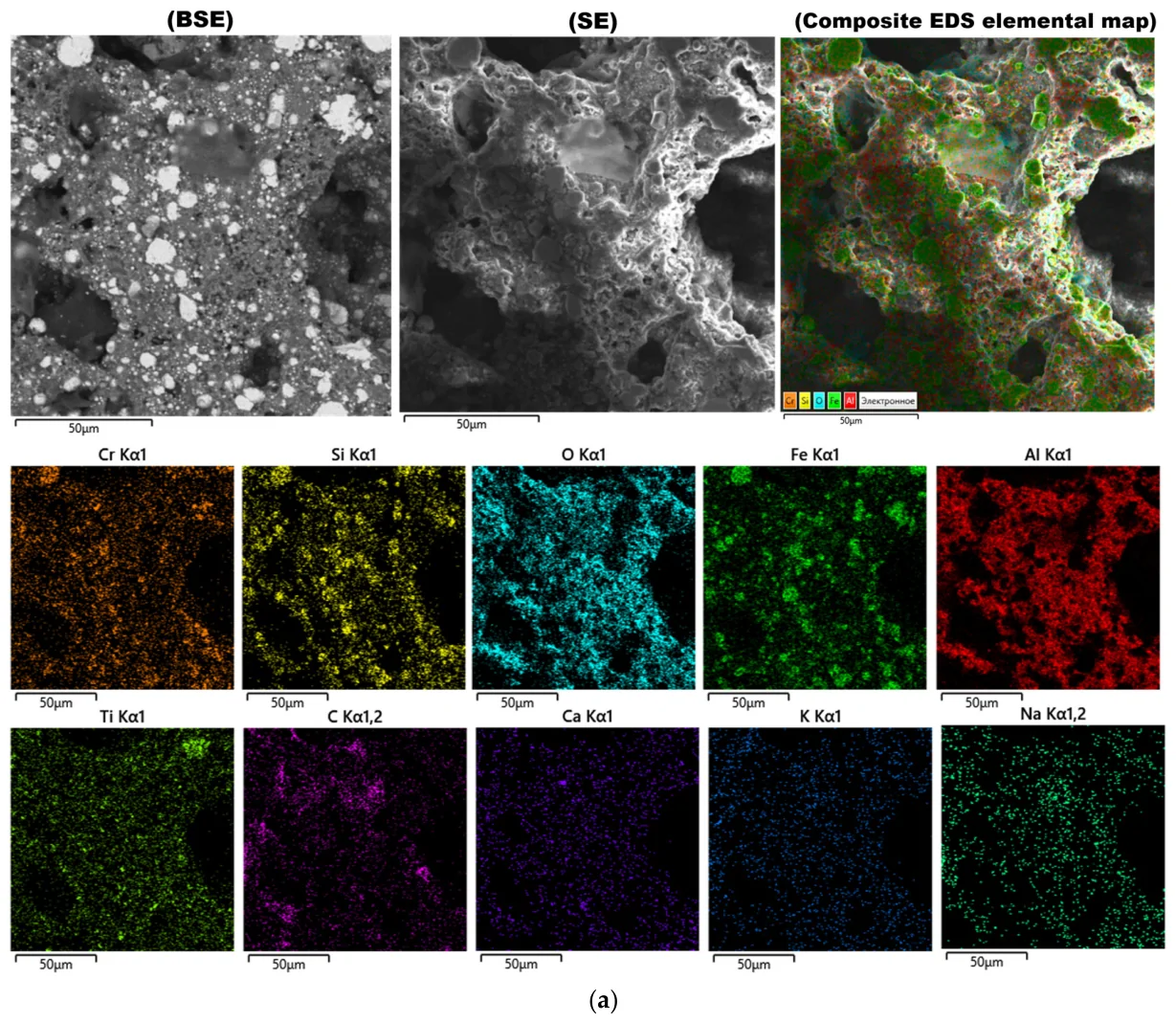

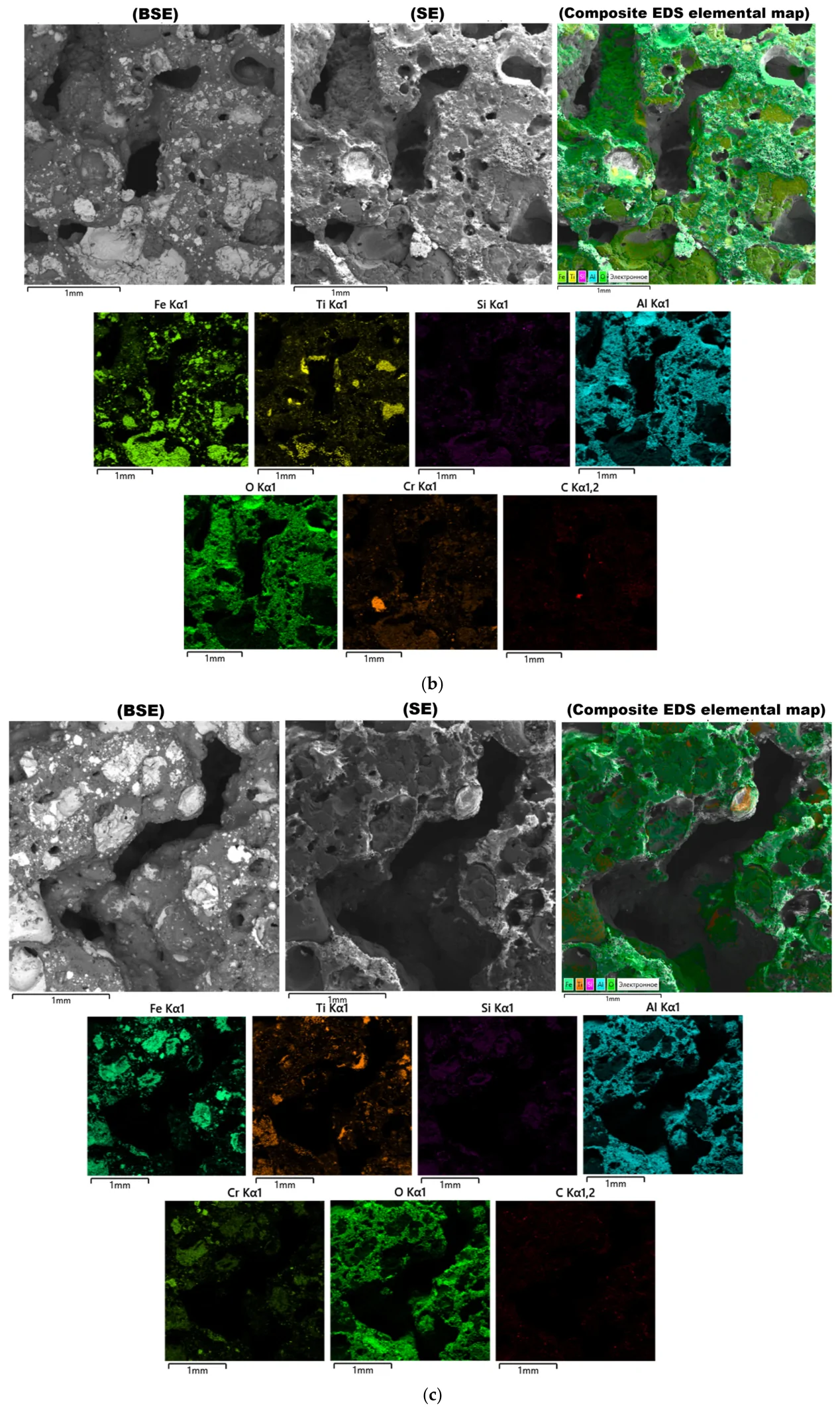
Microstructure of samples produced by SHS under air (**a**), argon (**b**), and vacuum (**c**) atmospheres: -backscattered electron (BSE) image; -secondary electron (SE) image; -superimposed EDS image with elemental distribution maps of Al, Fe, Si, Ca, Ti, Na, Cr, K, C, and O.

Figure 28a–c presents SEM images of the samples after SHS under vacuum, air, and argon atmospheres, respectively. Analysis of the micrographs reveals a well-developed porous structure characteristic of materials formed through rapidly propagating exothermic reactions. Pores with different sizes and morphologies are observed throughout the material. Their formation can be associated with gas evolution during SHS and the subsequent development of interconnected pore channels. Differences in pore morphology among the samples synthesized under different atmospheres are consistent with the experimentally determined variations in porosity and average pore diameter.

The secondary-electron (SE) images provide detailed information on the surface morphology and pore structure of the synthesized materials. The developed porous architecture can increase the specific surface area and may therefore influence the physicochemical and functional properties of the cermets. In addition, the distribution of the constituent phases and chemical elements within the solid framework may affect the mechanical behavior of the material; however, the individual contribution of each phase or element to the mechanical properties was not separately quantified in the present study.

The backscattered electron (BSE) image exhibits compositional contrast arising from variations in the average atomic number of the elements present in different regions of the sample. Brighter areas correspond to regions enriched with elements of higher atomic number, whereas darker areas are associated with the presence of lighter elements or pore spaces. The absence of sharply defined boundaries between contrasting regions suggests a relatively homogeneous distribution of the constituent phases and chemical components throughout the investigated material volume.

To determine the elemental composition of the synthesized material, energy-dispersive X-ray spectroscopy (EDS) analysis was performed. The resulting elemental distribution maps reveal the spatial localization of the constituent elements within the sample structure. The analysis indicates that the major elements are distributed relatively uniformly throughout the material volume, with no evidence of large-scale segregation regions. This observation suggests a high degree of reaction completion during the synthesis process and the formation of a homogeneous compositional structure.

The combined results of the SEM and EDS analyses confirm the formation of a porous material with a well-developed microstructure and a homogeneous distribution of chemical elements. The obtained data are consistent with the results of the X-ray diffraction (XRD) analysis and further verify the effectiveness of the selected self-propagating high-temperature synthesis (SHS) conditions for producing a material with the desired structural characteristics.

### 3.4. Mechanical Performance of SHS-Synthesized Porous Materials

**Figure 29:**
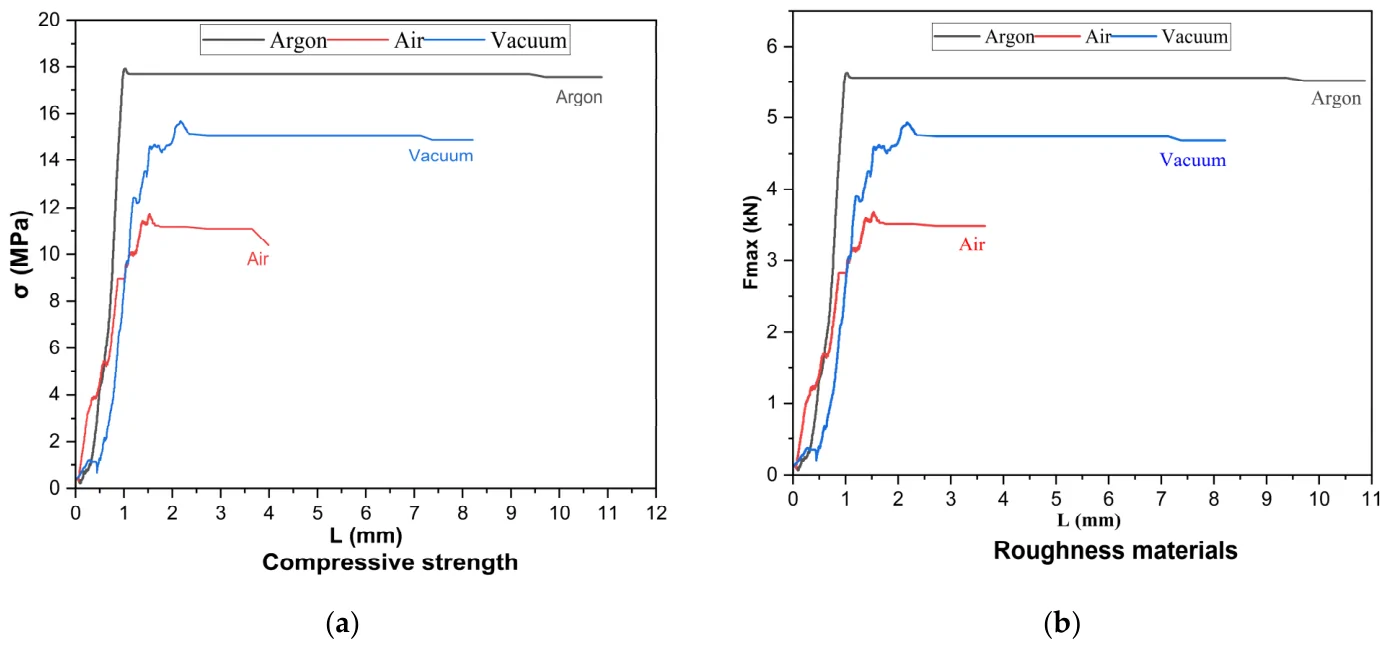
Compressive mechanical behavior of the S 4 porous cermet synthesized by self-propagating high-temperature synthesis (SHS) under different atmospheres (argon, vacuum, and air): (**a**) compressive strength and (**b**) load–displacement curves showing the deformation behavior up to failure.

Figure 29 shows the dependence of the compressive strength of a porous material produced by self-propagating high-temperature synthesis in three different environments: argon, air, and vacuum. As can be seen from the data presented, the highest compressive strength is observed during synthesis in an argon atmosphere—18 MPa; in air, it is slightly lower (11.9 MPa), whilst the lowest value is recorded under vacuum conditions (15.8 MPa). Figure 29b also shows the relationship between the maximum applied force and the amount of deformation, illustrating the degree of compression of the material up to its failure. Based on Hooke’s law, the stiffness coefficients of the materials were calculated as 0.53 for an argon atmosphere, 0.56 for air, and 0.61 for a vacuum, indicating differences in the elastic properties of the samples due to the synthesis conditions.

**Figure 30:**
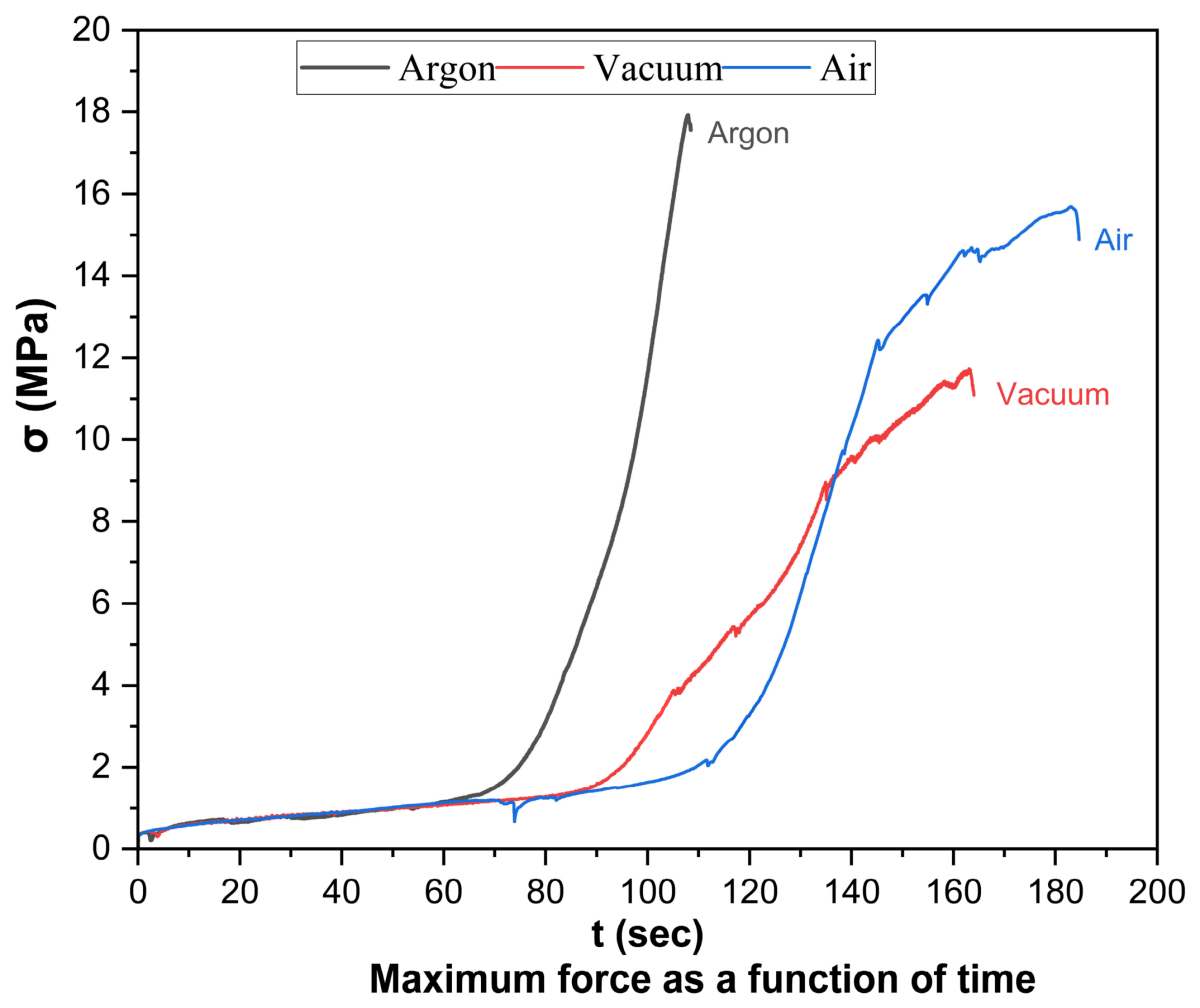
Comparison of the force–time response during compression of the S 4 porous cermet synthesized by self-propagating high-temperature synthesis (SHS) under different atmospheres (argon, vacuum, and air), showing the effect of the synthesis atmosphere on the load development and time required to reach the maximum load.

Figure 30 shows the relationship between the maximum applied force and time. Analysis of the graph indicates that the process of reaching the ultimate load occurs more rapidly for samples synthesized in argon than for those produced in air or a vacuum, which may be attributed to the characteristics of their microstructure and failure mechanism.

**Figure 31:**
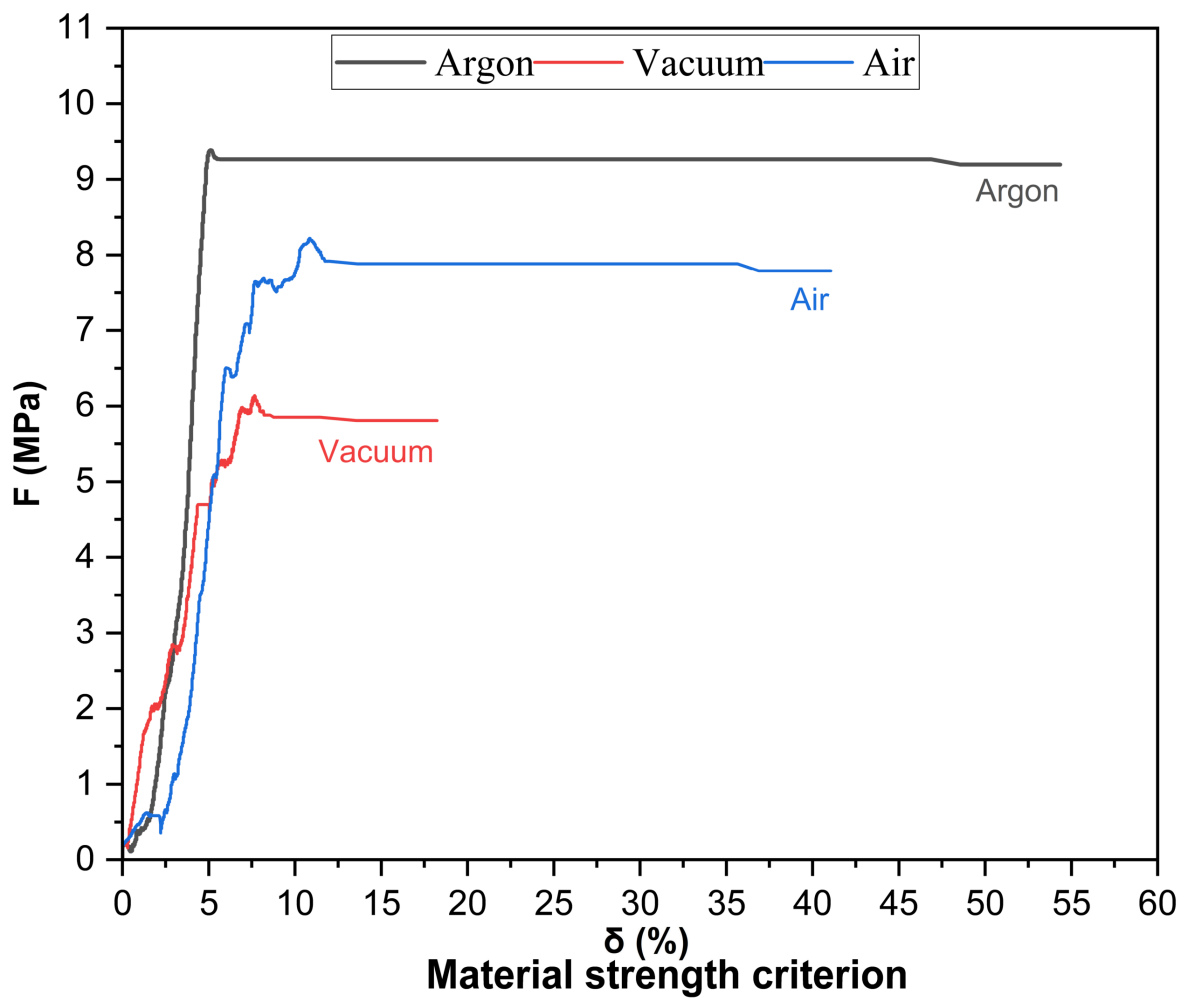
Comparison of the relative deformation (degree of compression) of the S 4 porous cermet synthesized by self-propagating high-temperature synthesis (SHS) under different atmospheres (argon, vacuum, and air), showing the effect of the synthesis atmosphere on the deformation behavior under compressive loading.

Figure 31 shows data on the relative deformation (degree of compression) of the material, expressed as a percentage. It has been established that maximum deformation is achieved during synthesis in argon (up to 50%), whereas in a vacuum it is around 30%, and in air it does not exceed 10%. This indicates greater plasticity and deformability in samples obtained in an inert atmosphere.

**Figure 32:**
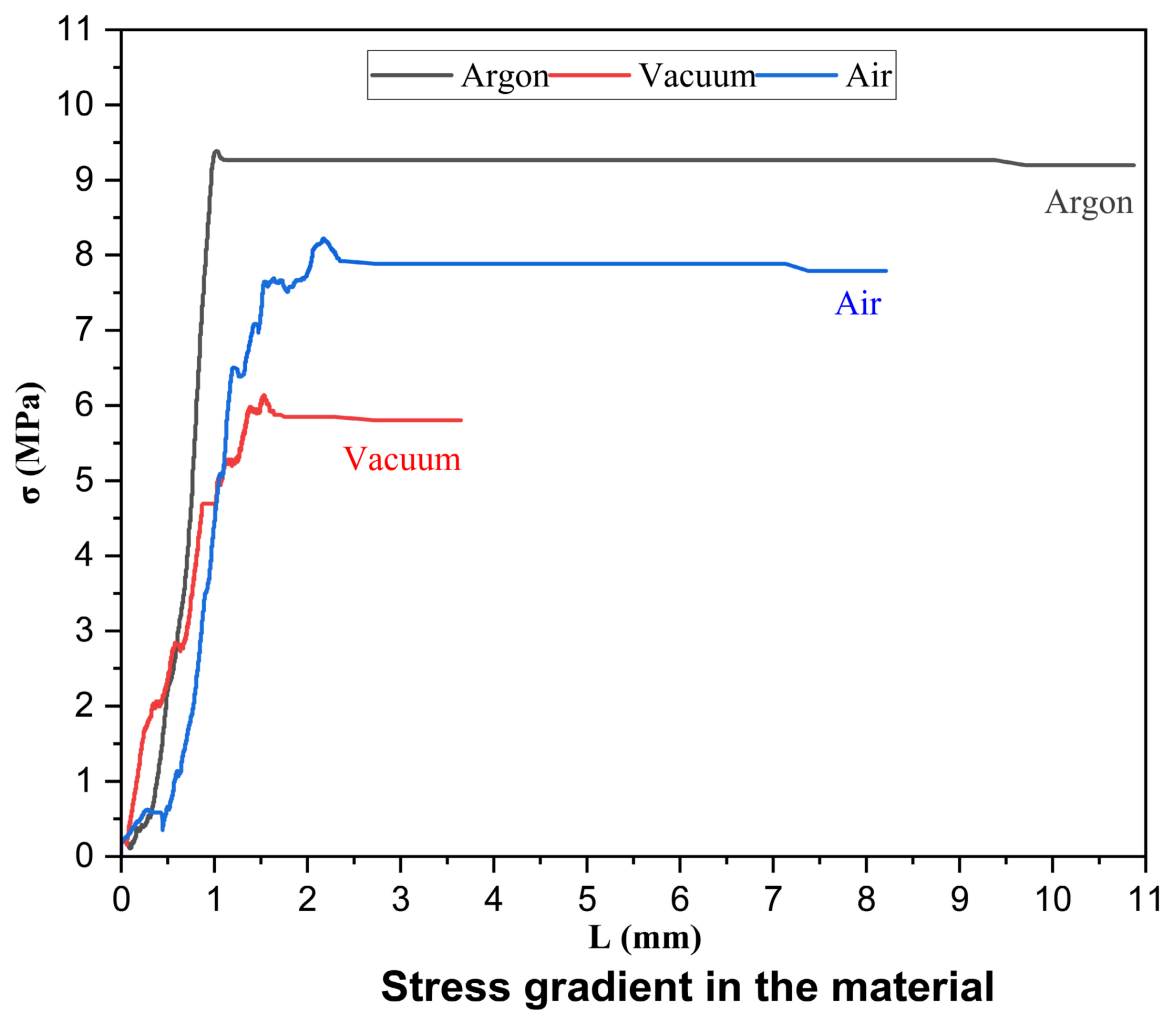
Comparison of stress development during compression of the S 4 porous cermet synthesized by self-propagating high-temperature synthesis (SHS) under different atmospheres (argon, vacuum, and air), showing the effect of the synthesis atmosphere on the stress response and mechanical behavior under compressive loading.

Figure 32 shows the dependence of the stress gradient on the material synthesis conditions. Analysis of the data obtained shows that the yield strength of the samples depends significantly on the environment in which the synthesis reaction took place: for samples obtained in an inert (argon) atmosphere, 9.5 MPa, in a vacuum, 7.9 MPa, and under air conditions, 5.9 MPa. This trend indicates a significant influence of the external environment on the formation of the material’s mechanical properties.

**Figure 33:**
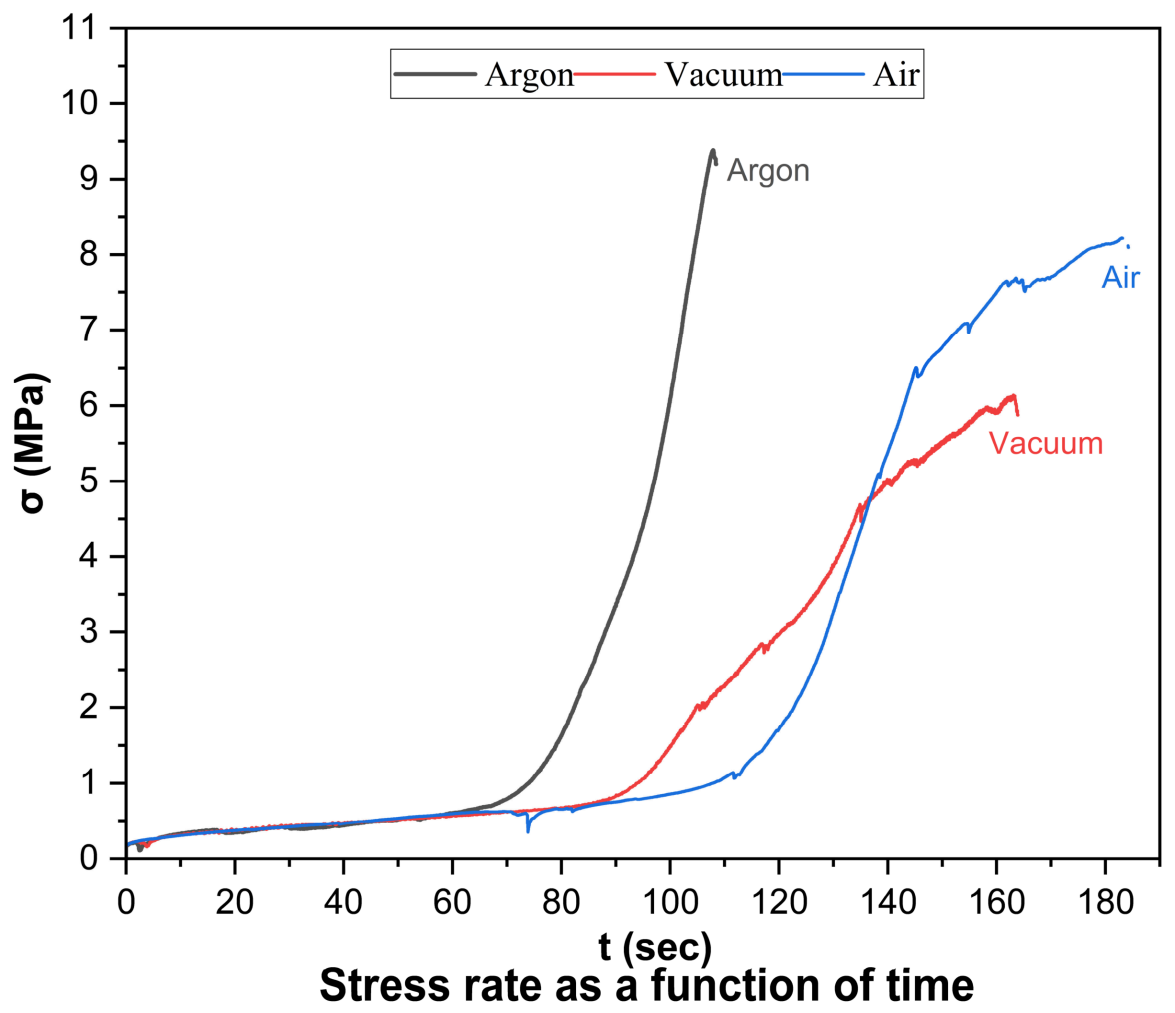
Comparison of the force–time response during compression of the S 4 porous cermet synthesized by self-propagating high-temperature synthesis (SHS) under different atmospheres (argon, vacuum, and air), showing the evolution of the applied force and the effect of the synthesis atmosphere on the compression behavior.

Figure 33 shows the dependence of the stress gradient on the material synthesis conditions. Analysis of the obtained data indicates that the yield strength of the specimens is significantly affected by the atmosphere in which the synthesis reaction was carried out. The yield strength was 9.5 MPa for the specimens synthesized in an inert (argon) atmosphere, 7.9 MPa for those synthesized in air, and 5.9 MPa for those synthesized under vacuum. This trend indicates a significant influence of the synthesis atmosphere on the development of the mechanical properties of the material.

Thus, it can be concluded that an increase in the stress gradient is accompanied by a decrease in the material’s deformation plasticity. This can be attributed to the fact that higher stress gradients promote more rapid accumulation of crystal-structure defects and localization of deformation processes. This conclusion is further supported by the analysis of the kinetics of stress variation with time. The rate of stress increase was found to be higher for the specimens synthesized in an argon atmosphere than for those synthesized in air and under vacuum. This indicates a more pronounced resistance of the material to external loading.

Consequently, an increase in the rate of stress change and the corresponding increase in yield strength result in improved strength characteristics of the material. At the same time, however, its ability to undergo plastic deformation decreases, resulting in increased brittleness. Therefore, the synthesis conditions represent an important factor governing the balance between strength and plasticity of the investigated material.

**Figure 34:**
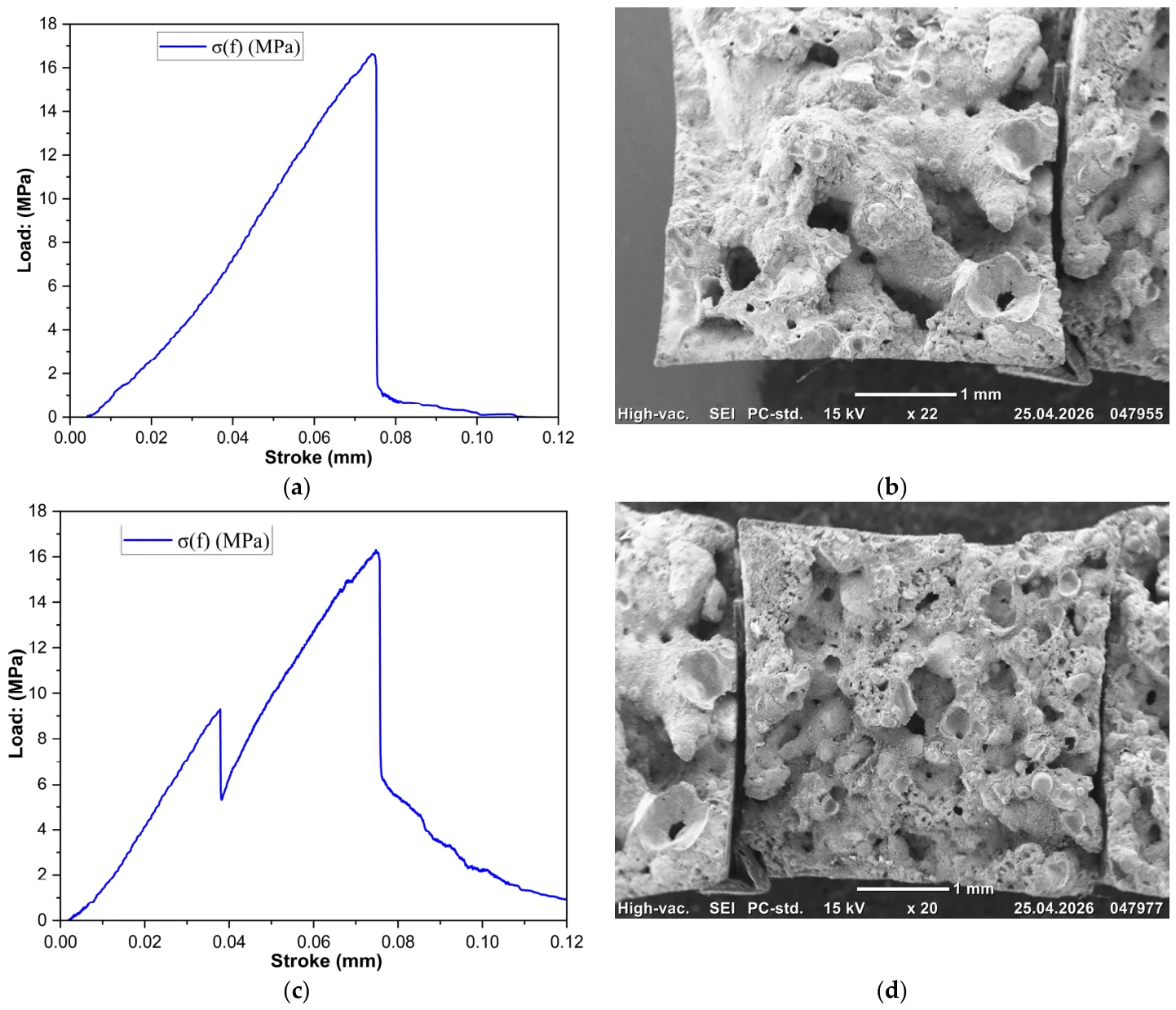

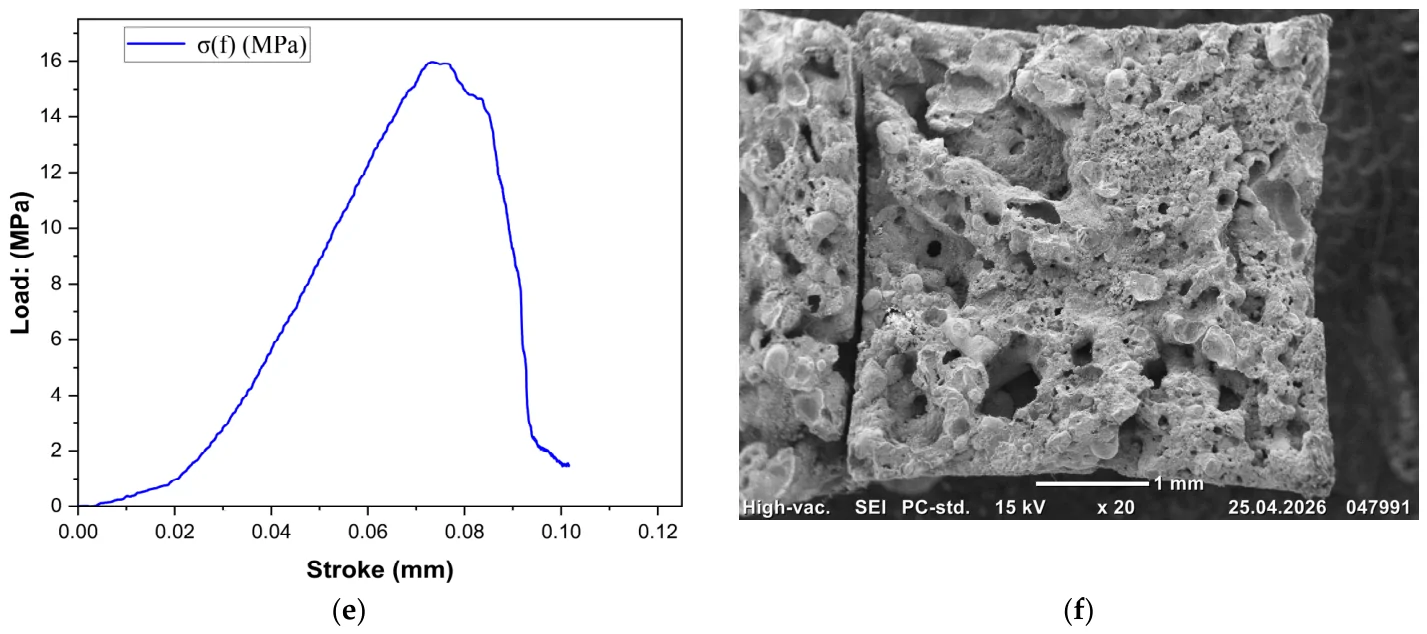
Comparison of the flexural behavior and fracture morphology of the S 4 porous cermet synthesized by self-propagating high-temperature synthesis (SHS) under different atmospheres: (**a**,**b**) vacuum, (**c**,**d**) air, and (**e**,**f**) argon. Panels (**a**,**c**,**e**) show the flexural strength and corresponding load–stroke curves, while panels (**b**,**d**,**f**) show SEM images of the corresponding fracture surfaces, illustrating the effect of the synthesis atmosphere on the flexural response and fracture characteristics.

Figure 34 shows the graphs of the flexural strength. As can be seen, the flexural strength of the material produced by self-propagating high-temperature synthesis in argon and air atmospheres was 17 MPa (see Figure 34e and c, respectively), and 16 MPa in a vacuum (see Figure 34a). In addition, the graph shows the material’s ability to resist cracking and withstand bending until failure: 0.18 mm in argon (see Figure 34e,f), 0.12 mm in air (see Figure 34c,d), and 0.12 mm in vacuum (see Figure 34a,b).

Table 9 presents the physical and mechanical properties of the obtained porous materials. Five specimens were tested for each synthesis condition, and the results are reported as mean values with the corresponding standard deviations (mean ± SD). Analysis of the results shows that the highest compressive strength was achieved for the specimens synthesized in an argon atmosphere, reaching 18 MPa. For the specimens synthesized under vacuum, the compressive strength was 15.8 MPa, whereas the lowest value, 11.9 MPa, was obtained for the samples synthesized in air.

In contrast, the highest elastic modulus was observed for the specimens synthesized under vacuum (53 GPa), while the values for the samples produced in argon and air were 36 GPa and 28.4 GPa, respectively. This difference is primarily attributed to the characteristics of the porous structure. Vacuum synthesis results in a material with lower total porosity and a smaller average pore size, thereby increasing its stiffness and resistance to elastic deformation. Conversely, the higher fraction of open porosity and the larger average pore diameter in the argon-synthesized specimens lead to a reduction in the elastic modulus.

Despite their higher porosity, the specimens synthesized in an argon atmosphere exhibited the highest compressive strength and yield strength. This indicates that the mechanical performance of the material is governed not only by the characteristics of the porous structure but also by its phase composition.

The observed physical and mechanical properties can be associated with the combined effects of preliminary mechanical activation, phase formation during SHS, and the resulting pore structure. Mechanical activation enhances the contact between the powder components and may promote aluminothermic reduction and subsequent reactions during SHS. XRD analysis confirmed the formation of Fe–Al and Cr–Al intermetallic compounds together with a Ti–Al–C phase. These phases may contribute to the mechanical strength and thermal stability of the solid framework; however, their individual contributions to the mechanical properties were not separately quantified in the present study.

The specimens synthesized under vacuum exhibited the highest elastic modulus, which can be associated with their lower porosity and smaller average pore diameter. In contrast, the specimens synthesized under argon showed a more developed porous structure with a higher fraction of open pores and a larger average pore diameter, resulting in a lower elastic modulus. Nevertheless, the argon-synthesized specimens maintained relatively high strength despite their increased porosity, indicating that the mechanical behavior is governed by the combined effects of pore structure and phase composition.

The mechanical test results further indicate that an increase in the stress gradient is accompanied by reduced deformation capacity. The rate of stress development was higher for the specimens synthesized under argon than for those produced in air and under vacuum, indicating a more rapid increase in resistance to the applied load. Overall, the results suggest that the synthesis atmosphere affects the balance between strength and deformation behavior through its influence on phase composition and pore structure.

## 4. Discussion

The present results demonstrate that the formation and performance of the SHS-derived porous cermets are controlled by the combined effects of the initial powder composition and the synthesis atmosphere. The investigated system differs from conventional porous ceramics because pore formation, reduction in oxide components, generation of a transient liquid phase, and formation of metallic and intermetallic constituents occur simultaneously during a rapidly propagating exothermic reaction. Consequently, the final porosity and mechanical properties cannot be interpreted solely in terms of the amount of an individual starting component; rather, they result from the balance between heat generation, gas evolution, liquid-phase redistribution, phase transformations, and subsequent solidification.

### 4.1. Effect of Powder Composition on Pore Formation

The variation in pore structure among the S 1–S 4 mixtures can primarily be associated with the different roles of Al_2_O_3_, Cr_2_O_3_, FeSi, and TiC during SHS. Al_2_O_3_ is a thermally stable constituent that does not directly contribute to the aluminothermic reduction of iron oxides and therefore acts mainly as a diluent and thermal regulator. Increasing its content reduces the effective heat release of the reactive mixture and can suppress excessive combustion temperatures and gas evolution. In contrast, Cr_2_O_3_ participates in the aluminothermic reaction:

Cr_2_O_3_ + 2Al → 2Cr + Al_2_O_3_ + Q, (14)

providing an additional source of heat. Therefore, variations in the Al_2_O_3_/Cr_2_O_3_ ratio influence the thermal balance of the SHS reaction and, consequently, the nucleation, growth, and coalescence of pores.

Pore formation in the present materials is associated with the rapid aluminothermic reaction of the oxide-containing mill scale with Al, accompanied by intensive heat release, phase transformation, and evolution of gaseous species. Gas cavities generated within the partially molten reaction zone become retained during rapid solidification, producing the characteristic porous framework. At the same time, the transient liquid phase can redistribute through the reacting compact and partially fill smaller voids. Thus, the final pore architecture reflects competition between gas-induced pore formation and liquid-phase-assisted pore filling and consolidation.

FeSi also plays an important role in this process. Its presence promotes the formation of a low-melting transient liquid phase and facilitates redistribution of Fe- and Si-containing species within the cermet skeleton. At the higher FeSi content used in S 1, this effect can increase liquid-phase mobility and modify pore connectivity. The experimental variations in porosity, permeability, and average pore diameter among S-1–S-4 therefore confirm that chemical composition provides an effective means of controlling the porous architecture of SHS-derived cermets.

This conclusion is consistent with previous studies showing that highly porous ceramic and cermet materials can be obtained through several different processing routes, including replication and direct foaming [27,28,29,30,31,32,33], spark plasma sintering [34,35,36,37], reactive processing [38,39,40,41,42], and carbothermal synthesis [43]. However, the mechanism of pore generation in the present SHS system differs fundamentally from template-based approaches because no sacrificial polymeric pore former is required. Instead, the porous structure develops directly as a consequence of the exothermic chemical reactions and gas/liquid-phase evolution occurring during SHS.

For comparison, Simonenko et al. [43] reported porous SiC ceramics with porosities of approximately 42–55% using carbothermal synthesis of SiO_2_–C precursors. In the present study, porosity values of a comparable or even higher magnitude were obtained directly from metallurgical waste-based reactive mixtures; for example, the S 4 material synthesized in argon exhibited a total porosity of approximately 60%, together with an average pore diameter of 121 μm and a permeability of 48%. This comparison shows that the SHS route used here can generate a highly developed porous structure without the prolonged thermal processing associated with conventional ceramic synthesis.

### 4.2. Effect of the Synthesis Atmosphere on Pore Structure and Phase Evolution

The synthesis atmosphere was found to be one of the most important parameters governing the final structure. A systematic tendency toward smaller pores and reduced porosity was generally observed under vacuum compared with synthesis in air and argon. This behavior can be explained by the reduced partial pressure of gaseous species under vacuum, which facilitates their removal from the reaction zone before extensive pore growth and coalescence occur. At the same time, the limited availability of atmospheric oxygen suppresses secondary oxidation reactions.

The opposite tendency is observed during synthesis in air. Oxygen can additionally react with Al and the reduced Fe-, Cr-, and Si-containing species during the high-temperature stage of SHS. These oxidation reactions modify the heat balance of the system and favor the stabilization of oxide phases. Additional local heat release and gas-related processes can promote the growth and coalescence of existing pores, producing a more open and coarser porous structure.

The experimental XRD results support this interpretation. The specimens synthesized in air exhibit a greater contribution from oxide phases, whereas the relative contribution of TiC and Fe–Al- and Cr–Al-containing reaction products decreases. Under vacuum and argon, suppression of secondary oxidation provides more favorable conditions for reduction reactions and for the preservation or formation of metallic, carbide, and intermetallic constituents. Thus, the observed changes in pore architecture are closely coupled with atmosphere-dependent phase evolution rather than being exclusively a physical consequence of gas-pressure differences.

This behavior is particularly relevant to Fe-containing oxide–Al systems. Previous investigations of porous Fe_2_O_3_–Al–Al_2_O_3_ and related materials [48,49,50,51,52,53,54,55,56] have demonstrated the suitability of aluminothermic reactions for producing Fe-containing porous structures. The present results extend this concept to alloy-steel mill scale containing a more chemically complex mixture of iron and alloying-element oxides and further demonstrate that the atmosphere can be used to control the competition between oxide stabilization and intermetallic-phase formation.

Previous investigations of Ni–Al and Ti–Al porous systems [46,47] have also demonstrated that intermetallic-forming reactions strongly affect the microstructure and functional properties of porous materials. However, the present Fe–Al-based approach offers an additional resource-efficiency advantage because the Fe-containing component is obtained from metallurgical waste rather than from exclusively high-purity metallic powders.

### 4.3. Role of TiC and Intermetallic Phases

The addition of plasma-synthesized TiC distinguishes the present system from many previously reported waste-derived SHS materials. TiC is a thermally stable and hard ceramic phase and can therefore act as a reinforcing constituent within the metallic/intermetallic skeleton. Studies of oxide-based ceramic matrices reinforced with TiC have previously demonstrated the importance of carbide additions for controlling hardness, flexural behavior, and fracture resistance [44], while other TiC-containing ceramic composites have shown that TiC can substantially modify both microstructure and mechanical response [45].

In the present cermets, TiC is incorporated into an Fe–Al-based reaction system rather than into a conventional dense ceramic matrix. Therefore, its function should be considered in combination with the phases generated in situ during SHS. XRD analysis indicates the formation of Fe–Al- and Cr–Al-containing intermetallic phases, while Ti-, Al-, and C-containing reaction products were also detected. Because the available diffraction data are insufficient to assign an unambiguous MAX-phase stoichiometry, these products are conservatively referred to here as a Ti–Al–C phase rather than being identified as a specific Ti_2_AlC or Ti_3_AlC_2_ compound.

The coexistence of TiC with Fe–Al- and Cr–Al-containing phases is important from the viewpoint of mechanical performance. The ceramic TiC particles can provide local resistance to deformation, whereas the metallic and intermetallic constituents provide structural continuity throughout the porous skeleton. This combination is consistent with the concept of metal–ceramic composites discussed in previous studies [19,20,21,22,23,24,25,26], in which ceramic reinforcement primarily contributes to hardness and thermal stability while the metallic component contributes to structural integrity and resistance to catastrophic brittle failure.

The SEM/EDS observations further support the formation of a multiphase porous framework. The principal elements are distributed relatively uniformly throughout the investigated regions, without evidence of pronounced macroscopic segregation. This suggests that preliminary mechanical activation followed by the rapid SHS reaction promotes effective contact and redistribution of the starting constituents. Nevertheless, because EDS mapping alone cannot establish the crystallographic identity of individual phases, the interpretation of the Fe–Al, Cr–Al, TiC, and Ti–Al–C constituents should be based primarily on the combined XRD and microstructural evidence.

### 4.4. Relationship Between Porosity, Phase Composition, and Mechanical Properties

The mechanical results reveal an important distinction between elastic stiffness and strength. The highest compressive strength was obtained for S-4 specimens synthesized in argon, reaching 18 MPa, whereas the corresponding values for vacuum and air were 15.8 and 11.9 MPa, respectively. The yield strength showed the same general tendency, with values of 17 MPa in argon, 15 MPa in vacuum, and 11 MPa in air. In contrast, the highest elastic modulus was measured for the vacuum-synthesized specimens (53 GPa), compared with 36 GPa in argon and 28.4 GPa in air.

The higher elastic modulus obtained under vacuum can be explained primarily by the reduced porosity and smaller pore dimensions. Pores decrease the effective load-bearing cross-section of a material and introduce regions of local strain concentration. Therefore, reduction in pore volume and pore size increases the resistance of the solid skeleton to elastic deformation. The vacuum-synthesized material is consequently stiffer even though it does not exhibit the highest ultimate compressive strength.

The higher compressive and yield strengths obtained in argon indicate that strength is not determined by porosity alone. In an inert atmosphere, secondary oxidation of the reduced metallic species is suppressed while the high-temperature SHS reaction can proceed without direct interaction with atmospheric oxygen. These conditions favor the preservation and formation of TiC and Fe–Al-, Cr–Al-, and Ti–Al–C-containing constituents within the load-bearing skeleton. The resulting multiphase framework can therefore withstand higher compressive loads despite having a more developed porous structure than the vacuum-synthesized material.

The lower compressive strength observed after synthesis in air is consistent with both the phase and structural results. Increased oxidation reduces the relative contribution of metallic/intermetallic load-bearing regions, while larger and more developed pores reduce the effective cross-sectional area and act as local stress concentrators. These factors facilitate crack initiation and propagation and explain why the air-synthesized specimens exhibited the lowest compressive and yield strengths.

This interpretation is supported by the flexural tests. The flexural strength was 17 MPa for specimens synthesized in argon and air and 16 MPa for those produced under vacuum. However, the displacement before failure reached 0.18 mm for the argon-synthesized material compared with 0.12 mm for both air and vacuum. This suggests that the argon-derived structure provides a favorable compromise between interconnected porosity and integrity of the load-bearing multiphase framework.

The absolute strength values obtained in this work are lower than the compressive strengths of approximately 53–72 MPa reported for porous intermetallic materials produced by low-pressure reactive processing at 700–900 °C [38,39,40,41,42]. However, a direct comparison should be made cautiously because the processing route, starting materials, phase composition, and especially porosity are substantially different. The present materials were intentionally designed as highly porous and permeable cermets, with the S-4 argon condition reaching approximately 60% porosity. A reduction in mechanical strength is therefore expected compared with denser intermetallic materials. More importantly, the present approach provides a combination of open porosity, permeability, and mechanical integrity while using metallurgical waste as the principal Fe-containing raw material.

This comparison also emphasizes that maximum strength was not the sole objective of the present study. Porous permeable components intended for filtration, catalyst support, and high-temperature flow applications require a balance among interconnected porosity, permeability, thermal stability, and sufficient mechanical integrity [17,18]. Therefore, the S-4 material synthesized in argon is of particular interest because it combines a developed porous structure (approximately 60% porosity, 121 μm average pore diameter, and 48% permeability) with the highest measured compressive strength of 18 MPa and yield strength of 17 MPa among the investigated synthesis atmospheres.

### 4.5. Significance of the Waste-Derived SHS Route

The present results are generally consistent with earlier studies showing that metallurgical residues can be converted into porous functional materials [14,15,16] and with previous investigations of Fe-containing oxide–Al SHS systems [48,49,50,51,52,53,54,55,56]. They also support earlier work demonstrating the feasibility of producing porous permeable cermets from metallurgical wastes by SHS [57,58,59,60,61]. However, the present study extends these approaches by combining alloy-steel mill scale with plasma-synthesized TiC, FeSi, and oxide additives and, importantly, by systematically examining the effect of air, argon, and vacuum environments on phase evolution, pore development, and mechanical behavior.

Accordingly, the principal outcome of this study is not simply the production of another porous SHS material but the establishment of a processing–structure–property relationship for a waste-derived TiC-reinforced Fe–Al-based cermet. The results show that the atmosphere provides an effective tool for tailoring the balance between porosity and mechanical behavior: vacuum promotes a finer and denser pore structure and the highest elastic stiffness, argon provides the highest compressive and yield strengths while maintaining high open porosity and permeability, and air promotes oxidation and pore coarsening, resulting in reduced compressive performance.

These findings demonstrate that no single synthesis atmosphere is universally preferable; instead, the processing conditions should be selected according to the targeted application. A denser and stiffer structure may favor vacuum processing, whereas argon is more suitable when a combination of high permeability, developed open porosity, and mechanical strength is required. Further work should quantify the individual phase fractions and their contributions to mechanical behavior and should evaluate long-term oxidation resistance, corrosion behavior, thermal cycling stability, and permeability under service conditions before application-specific performance can be established.

## 5. Conclusions

1. A comprehensive technological route for producing porous cermets from alloy steel scale by self-propagating high-temperature synthesis (SHS) was successfully developed. The proposed approach includes the synthesis of titanium carbide powder by a non-vacuum electric arc plasma method, mechanical activation of reactive powder mixtures, SHS under various atmospheres, and comprehensive structural, phase, and mechanical characterization of the synthesized materials.
2. The non-vacuum electric arc plasma method enabled the successful synthesis of titanium carbide powder suitable for incorporation into the SHS charge. X-ray diffraction confirmed the predominant formation of the TiC phase (96 wt.%), while SEM analysis revealed the characteristic morphology of the synthesized particles. The obtained TiC powder effectively served as a reinforcing ceramic component in the porous cermets.
3. Mechanical activation was found to play a decisive role in increasing the homogeneity and reactivity of the powder mixtures. Within the investigated range, a milling speed of 800 rpm and a duration of 40 min provided the most favorable conditions for subsequent SHS reactions. Prolonged activation to 50 min resulted in particle agglomeration caused by intensive plastic deformation.
4. The synthesis atmosphere significantly affected the phase composition, pore morphology, and mechanical performance of the porous cermets. Within the investigated range, the most favorable combination of properties was obtained under an argon atmosphere for the composition FeO-45 wt.%, Al-20 wt.%, Al_2_O_3_-7.5 wt.%, Cr_2_O_3_-5 wt.%, TiC-10 wt.%, and FeSi-12.5 wt.%, which exhibited a total porosity of 60%, an average pore diameter of 121 μm, and a permeability of 48%. These results demonstrate that the pore structure can be effectively tailored through appropriate control of the SHS processing conditions.
5. SEM/EDS observations and X-ray diffraction analysis confirmed the formation of a well-developed interconnected porous structure and identified Fe-Al and Cr-Al intermetallic phases together with a Ti-Al-C phase in the SHS-derived materials, indicating the progression of high-temperature reactions within the reactive powder mixture. The presence of these phases may contribute to the mechanical strength and thermal stability of the porous material at elevated temperatures; however, the individual contribution of each phase to the mechanical properties was not separately quantified in the present study. In addition, oxide phases were identified in the synthesized materials, coexisting with the intermetallic phases and resulting in a complex multiphase microstructure.
6. Mechanical testing demonstrated that the synthesized porous cermets possess a favorable combination of structural integrity and mechanical performance. The materials exhibited a compressive strength of 18 MPa, a flexural strength of 17 MPa, a yield strength of 17 MPa, an elastic modulus of 36 GPa, and a hardness of 860 HV, confirming their suitability for engineering applications requiring lightweight porous structures.

Overall, the present study demonstrates an effective route for converting large-tonnage alloy steel scale from metallurgical waste into high-value porous cermets with controllable structural and mechanical properties. The developed materials show considerable potential for filtration systems, catalyst supports, thermal insulation, and other high-temperature engineering applications. Long-term oxidation resistance, corrosion behavior, and service performance were beyond the scope of the present study and therefore represent its main limitations. Future research will focus on evaluating these properties and further optimizing the chemical composition of the reactive mixtures.

## Figures and Tables

**Table 1:** Average particle sizes of the initial powders used for the preparation of the SHS powder mixtures.

Chemical Elements	Mechanical Engineering Waste (±5 mm)	Aluminum (±1 µm)	Aluminum Oxide (±0.5 µm)	Chromium Oxide (±0.5 µm)	Ferrosilicon (±0.5 µm)
Size	20 mm	30 µm	10 µm	4 µm	7 µm

**Table 2:** Compositions of the S 1–S 4 powder mixtures and corresponding mechanical activation parameters.

Number of Samples *	Composition						Time (min)			Experimental Conditions	Rotational Speed (rpm)
	Scale	Al	Al_2_O_3_	Cr_2_O_3_	TiC	FeSi					
1 (S 1)	40	20	7.5	5	10	17.5	30	40	50	Air	800
2 (S 2)	40	20	7.5	7.5	12.5	12.5					
3 (S 3)	40	20	10	5	12.5	12.5					
4 (S 4)	45	20	7.5	5	12.5	12.5					

* The powder mixtures are denoted as S 1 to S 4.

**Table 3:** Processing parameters used for the self-propagating high-temperature synthesis (SHS) of the porous cermet samples.

Number of Samples	Arc Voltage (V)	Current (A)	Synthesis Atmosphere (Pressure, kgf/cm^2^)		
			Vacuum	Air	Argon (Ar)
1 (S 1)	110	10	−0.9	0	0.1
2 (S 2)					
3 (S 3)					
4 (S 4)					

**Table 4:** Analytical results for titanium carbide (TiC) powder synthesized by the non-vacuum electric arc method in air.

Anode Weight, g		Weight Inside the Crucible, g		Weight of the Crucible Lid, g		Weight of the Outer Crucible, g		Weight of the Mixture, g	Weight of the Product Obtained	Yield, %	Current	Time	Graphite Paper, g		Total Mass, g	
Until	After	Until	After	Until	After	Until	After						Until	After	Until	After
81.1	78.67	102.4	106.56	41.19	38.79	243.16	239.27	90.32	39.3	43.5	300	300	1.37	1.37	479	424
71.1	69.41	93.88	93.59	38.79	35.73	238.33	232.1	38.18	35.5	96	300	300	1.37	1.37	414.82	399.44

**Table 5:** Comparison of the average particle size of the S 1 powder mixture after mechanical activation at 800 rpm for different activation times (30, 40, and 50 min).

Time, min	30	40	50
Average particle size, µm (±0.1 µm)	7.4	5.8	1.3

**Table 6:** Comparison of the average particle size of the S 2 powder mixture after mechanical activation at 800 rpm for different activation times (30, 40, and 50 min).

Time, min	30	40	50
Average particle size, µm (±0.1 µm)	6.8	4.4	2.5

**Table 7:** Comparison of the average particle size of the S 3 powder mixture after mechanical activation at 800 rpm for different activation times (30, 40, and 50 min).

Time, min	30	40	50
Average particle size, µm (±0.1 µm)	6.4	5.5	3.1

**Table 8:** Comparison of the average particle size of the S 4 powder mixture after mechanical activation at 800 rpm for different activation times (30, 40, and 50 min).

Time, min	30	40	50
Average particle size, µm (±0.1 µm)	6.9	4.2	2.4

**Table 9:** Comparison of the mechanical properties of the S 4 porous cermet synthesized by self-propagating high-temperature synthesis (SHS) under different atmospheres (argon, vacuum, and air).

Mechanical Characteristics Mean ± SD	Argon	Vacuum	Air
F_max_ (kN) (±0.7)	5.63	3.68	4.92
σ (MPa) Compressive strength (±1.4)	18	15.8	11.9
E GPa Modul of elasticity (±5.8)	36	53	28.4
σ_T_ MPa Yield strength (±6.8)	17	15	11
σ_b_ MPa Bending strength (±5.7)	17	16	17
Hardness (HV) (±10.5)	860	859	870

## Data Availability

The original contributions presented in this study are included in the article. Further inquiries can be directed to the corresponding authors.
